# Unraveling Plant Natural Chemical Diversity for Drug Discovery Purposes

**DOI:** 10.3389/fphar.2020.00397

**Published:** 2020-04-07

**Authors:** Emmanuelle Lautié, Olivier Russo, Pierre Ducrot, Jean A. Boutin

**Affiliations:** ^1^ Centro de Valorização de Compostos Bioativos da Amazônia (CVACBA)–Instituto de Ciências Biológicas, Universidade Federal do Pará (UFPA), Belém, Brazil; ^2^ Institut de Recherches Internationales SERVIER, Suresnes, France; ^3^ Molecular Modelling Department, 'PEX Biotechnologie, Chimie & Biologie, Institut de Recherches SERVIER, Croissy-sur-Seine, France

**Keywords:** secondary metabolites, drug production, natural products, renewal sources, plant chemodiversity, synthetic biology, *in vitro* cultures, medicinal chemistry

## Abstract

The screening and testing of extracts against a variety of pharmacological targets in order to benefit from the immense natural chemical diversity is a concern in many laboratories worldwide. And several successes have been recorded in finding new actives in natural products, some of which have become new drugs or new sources of inspiration for drugs. But in view of the vast amount of research on the subject, it is surprising that not more drug candidates were found. In our view, it is fundamental to reflect upon the approaches of such drug discovery programs and the technical processes that are used, along with their inherent difficulties and biases. Based on an extensive survey of recent publications, we discuss the origin and the variety of natural chemical diversity as well as the strategies to having the potential to embrace this diversity. It seemed to us that some of the difficulties of the area could be related with the technical approaches that are used, so the present review begins with synthetizing some of the more used discovery strategies, exemplifying some key points, in order to address some of their limitations. It appears that one of the challenges of natural product-based drug discovery programs should be an easier access to renewable sources of plant-derived products. Maximizing the use of the data together with the exploration of chemical diversity while working on reasonable supply of natural product-based entities could be a way to answer this challenge. We suggested alternative ways to access and explore part of this chemical diversity with *in vitro* cultures. We also reinforced how important it was organizing and making available this worldwide knowledge in an “inventory” of natural products and their sources. And finally, we focused on strategies based on synthetic biology and syntheses that allow reaching industrial scale supply. Approaches based on the opportunities lying in untapped natural plant chemical diversity are also considered.

## Background on Natural Compounds in Drug Discovery

### Drugs and Natural Products

Several reviews, like the updated survey from Newman and Cragg (2016), pointed to the fact that many drugs on the market are from natural origin; these authors stated that, out of the 1,328 new chemical entities approved as drugs between 1981 and 2016, only 359 were purely of synthetic origin. From the remaining ones, 326 were “biological” entities (peptides of more than 50 residues, including therapeutic antibodies), and 94 were vaccines. A little less than half of those new drugs (549, exactly) were from natural origin or derived inspired from natural compounds. Furthermore, in the anticancer area, out of the 136 approved nonbiological compounds from the same period (1981–2014), only 23 were purely synthetic (*i.e.* not derived from natural compounds nor natural compounds themselves) (Newman and Cragg, 2016). Natural origin can have different definitions, and these authors accounted for three categories: unaltered natural (pure) products; defined mixture of natural products (NP) and natural product derivatives isolated from plants or other living organisms as fungi, sponges, lichens, or microorganisms; and products modified by medicinal chemistry. There are many examples: anticancer drugs such as docetaxel (Taxotere™), paclitaxel (Taxol™), vinblastine, podophyllotoxin (Condylin™), or etoposide; steroidal hormones such as progesterone, norgestrel, or cortisone; cardiac glycosides such as digitoxigenin; antibiotics like penicillin, streptomycin, and cephalosporins [see IA Ross for more examples (1999)]. Furthermore, Rodrigues et al. (2016) pointed to the fact that fragments derived from natural structures are a source of diverse molecules from which new drugs can be designed, thanks to the fragment-based drug discovery approach (Erlanson et al., 2016; Mortenson et al., 2018; Yñigez-Gutierrez and Bachmann, 2019).

### Screening for New Drugs and Discovery Approaches

Besides the understanding of pathological processes, the source of molecules has been a main concern for the pharmaceutical industry. Vast libraries of compounds have been established in order to feed the research. For example, in midsize pharmaceutical companies, it is common to find libraries from 30,000 up to 500,000 compounds, while for big pharmas, the numbers are more in the 500,000 to several million ranges (Macarron et al., 2011). To our knowledge this is also the case for the National Chinese Compound Library in Shanghai, China (http://en.cncl.org.cn/). Finally, national or transnational efforts have been reported to create such depositories of compounds for the use of screening programs from the Academy: see Horvath et al. (2014) in Europe and Thornburg et al. (2018) for the NIH/NCI effort. In addition, vendors are also selling libraries of compounds composed of a “large” diversity that they build according to different principles (Boss et al., 2017). Several publications deal with how the compounds are chosen (Langer et al., 2009), if they are following Lipinski rules (Lipinski et al., 2001; Lipinski, 2003) or not, if they are virtual (Glaab, 2016) or real, if they are systematically tested on all the targets, how they can be organized in subclasses of compounds designed to potentially interact with channels, receptors, or enzymes, *etc*. Furthermore, the composition of the library in relation with the main categories of molecules—small synthetic compounds, drug-like organic compounds, peptides, proteins, sugars, nucleosides, or natural compounds—can greatly vary in function of the “Pharma company culture”, that is to say, the compounds that have already been synthesized in a given company as well as the “sensitivity” of the medicinal chemists and screening people.

When the decision to incorporate natural products is made, pure well-defined compounds or extracts are selected according to different criteria: pharmacognosy, ethnopharmacology, or even traditional knowledge. Because of the traditions existing in the uses of botanicals and medicinal plants, this empirical knowledge has accumulated for ages and passed through generations. Modern pharmacology has explored and validated probably only a minor part of this knowledge through attempts of rationalizing the use of plants as sources of drugs. This first possible approach would be a way to guide some drug discovery projects. Another totally different approach based on the use of high-throughput screening (HTS) emerged one or two decades ago. It aimed at exploring systematically the immense chemical diversity in secondary metabolites and was based on the technological developments of discovery tools such as miniaturization and automation (Atanasov et al., 2015). In this sense, the increase of the amount of compounds was tested simultaneously, and the scientific rationalization of the selection of those compounds, thanks to the growing capacities of chemo-informatic approaches (Larsson et al., 2005; Larsson et al., 2007), emerged as trends in HTS.

### Strategies for Identification of Bioactive Compounds From Crude Extracts

When reviewing the literature on how discovery of plant-derived actives is performed, the following pattern emerges (Weller, 2012; Sharma and Gupta, 2015, Heinrich et al., 2018). Plants are collected in precise geo-localized sites. The collection may include the whole plant or any part such as leaves, stems, bark, seeds, or roots. Then, the botanical material is dried and powdered using mechanical means, such as grounders. Those powders are then extracted with solvents at different temperatures or of increasing polarities with sequential extraction procedures in the cases when, for example, the chemistry of the active compounds is unknown. These first steps are important to consider as the extraction method might influence the chemical composition of the extract and consequently, its biological activity.

Then, the extracts are dried under low pressure, and the final solid residue is suspended in a solution comprising the minimal amount of a biological-compatible solvent, often DMSO. The next steps would include the testing of the extracts in 96 (or 384) well plates against the biological targets of the program. These biological tests are often a cloned enzyme catalytic assay, a receptor binding assay, a protein–protein interaction assay or even a whole pathway (Ishibashi and Ohtsuki, 2008; Xie et al., 2018), to name but a few. Biological testing targeting whole-cells was also reported against cancer cells (Mazzio et al., 2014; Kant et al., 2016), virus-infected cells (Yi et al., 2004; Dai et al., 2012), or microorganisms (Correia et al., 2008; Figueroa-López et al., 2014; St-Pierre et al., 2018). Extracts showing activity on those tests are selected and submitted to fractionation by chromatography. Each fraction (typically around ten) will be tested in turn in the same assay, and the active fractions are subjected to one or several extra fractionations, often using alternative chromatographic conditions.

Some of these experiments are based on an HTS environment. HTS methodologies provided us with impressive progresses in terms of increased speed of assay and lowering of price. Indeed, robots can handle several thousands of tests during a workday. However, the initial enthusiasm for HTS (Harvey and Cree, 2010; Sarker and Nahar, 2012) when applied to crude extract libraries in targeted assays systems has been facing several issues as stated recently by Thornburg et al. (2018). In fact, HTS techniques do not really modify the discovery process itself. For example, starting with 2,000 extracts—which is a modest number considering (a) the number of plants species available and (b) the number of compounds in an HTS campaign—might result into 10% actives, in the best case. Thus, the next step would be 200 hydrophobic columns, with the collection of about 10 fractions per chromatography, and then 2,000 tests. As exemplified, the real bottleneck in this whole process is the parallel fractionations of the actives; as of today, only partial progresses in their automatization have been reported over the last years (Sharma and Gupta, 2015).

Other strategies have been developed that delivered results. In their detailed review, Atanasov et al. (2015) classify into five groups the strategies to identify bioactive compounds from plant extracts: the bioactivity-guided fractionation strategy previously mentioned, the similar synergy-directed fractionation strategy, the metabolic profiling strategy, the metabolism-directed (biotransformation focused) strategy, and finally the direct phytochemical isolation strategy. The group of metabolic profiling approaches has a more detached relationship with bioactivity as they are not focused on compounds. They were developed during the last 10–15 years in plant natural products. Using highly sensitive and reproducible analytical methods, they allow the correlation between chemical profile (qualitative or quantitative) and bioactivity data (Zampieri et al., 2017; Parrot et al., 2018; Wolfender et al., 2019) with recent progress in the field of data analysis and integration at the extract level (Wolfender et al., 2019). Finally, the group of direct phytochemical isolation approaches focuses on the comprehensive chemical characterization of the plant extract and the isolation of novel scaffolds without immediately evaluating their bioactivity.

### Successes and Limitations

Some of the successes in terms of new drug development have already been mentioned in *Drugs and Natural Products.* But in terms of screening and strategy for finding an active compound in an extract as an enzyme inhibitor or a protein/protein interaction inhibitor, many successes have also been reported. Some examples of screening results of extracts with those approaches (Atanasov et al., 2015) can be mentioned here, but being exhaustive is impossible, as literally hundreds of such tests were performed [*e.g*. from our laboratories (Bousserouel et al., 2005; Lautié et al., 2008; Litaudon et al., 2009a; Litaudon et al., 2009b; Columba-Palomares et al., 2015; Catteau et al., 2017; Olivon et al., 2018) and from hundreds of others]. Some examples reported recently are given in **Table 1** of the inhibitory activities of plant extracts on specific enzymes. Of note, the panel of enzyme activities is large and diverse; the examples in **Table 1** reflect the current trend in terms of enzyme of origin for this type of work: they are mainly coming from cancer and diabetes/obesity fields. It is important to say that an unknown number of failing attempts remain unpublished.

**Table 1:** Some examples of enzyme inhibiting extracts from natural origin.

Enzyme	Plant extract	Reference
Carbohydrase	*Hibiscus sabdariffa*	(Ifie et al., 2016)
Cholinesterases	*Jatropha gossypifolia*	(Saleem et al., 2016)
COX-1, COX-2	*Onopordum acanthium*	(Lajter et al., 2015)
Cytochrome P450 3A	*Aframomum melegueta, Dennettia tripelata*	(Nduka et al., 2017)
Cytochrome P450 1B1	*Glycyrrhiza glabra*	(Sharma et al., 2017)
DNA polymerase	*Sorbus acuparia*	(Turumtay et al., 2017)
Elastase	*Tagetes erecta*	(Vallisuta et al., 2014)
Lipoxygenase, urease	*Polygonatum verticillatum*	(Khan et al., 2015)
Metalloproteinase	*Citrus paradisii, Citrus sinensis, Citrus maxima*	(Ademosun et al., 2015)
Monoamine oxidase A	*Hypericum perforatum, Paganum harmal*	(Herraiz and Guillen, 2018)
Angiotensin converting enzyme	*Vernonia amygdalina, Gongronema latifolium*	(Ajibola et al., 2011)
N-myriostoyltransferase	*Terminalia bentzoë*	(Apel et al., 2018)
Protein tyrosine phosphatase	*Eugenia jambolana*	(Liu et al., 2017)
Tyrosinase	*Gynotroches axillaris*	(Abed et al., 2016)
Tyrosinase	*Phaleria macrocarpa*	(Nadri et al., 2014)
Urease	*Rumex nervosus*	(Khan et al., 2014)
Xanthine oxidase	*Fraxinus angustifolia, Pistacia lentiscus*	(Berboucha et al., 2010)
*α*-Amylase	*Urtica dioica, Juglans regia*	(Rahimzadeh et al., 2014)
*α*-Glucosidase	*Terminalia macroptera*	(Pham et al., 2014)
*β*-ketoacyl-ACP reductase	*Alpinia officinarum*	(Huang et al., 2008)
Xanthine oxidase	Puertorican plants*	(Guerrero and Guzman, 1998)
Epoxide hydrolase	Chinese herbs*	(Shi et al., 2008)
HIV-1 integrase	Chinese herbs*	(Au et al., 2001)
Acetylcholinesterase	Marine fungi**,*	(Liu et al., 2014)
Lipase	*Chlorella* sp.***	(Sibi, 2015)
Lipase	Ascophyllum nodosum***	(Austin et al., 2018)

*: unspecified genuses; **: fungi; ***: algae

For the last thirty years screening for new molecules—whether candidate drugs *per se* or hit compound that should be modified—is one of the two main sources of drugs for the pharmaceutical industry. These screening strategies delivered mixed results, and several factors complicated dramatically the analyses of these results. The main ones are in our opinion: the way diseases can be simplified—or not—to a molecular target that is amenable to HTS; the choices of the molecules which can be screened into these assays; the quality of the assays and/or their sensitivity; the statistical considerations to determine the threshold at which a compound is recognized as an active; and finally, the source of the screened molecules. We (Boutin et al., 2018) and others (Schmid et al., 1999; Dufresne, 2000; Thiericke, 2000; Mayr and Bojanic, 2009; Bodle et al., 2017) exposed our screening strategies at several occasions, and considering the diversity of the potential problems, it can be hard to identify a universal approach. This is a fact that HTS promising techniques have not delivered as many drugs as expected with regard to the price and the ambition of the solutions/organizations involved (Newman and Cragg, 2016).

#### Several Limitations in the “Classical” Strategies to Identify Bioactive Natural Products

The first type of limitation would be related to the collections of living organisms and access to plant species, for example. The probability still exists that a plant collected on a given location would not be there the next time around, especially the whole plant. If bioactivities associated with this species are discovered during the process, it could be hard to find again the same plant population. In those cases, two choices are possible: to look for this species in another location or to look in the same location for a closely related species with the help of botanists. Neither solution is entirely satisfactory: the change of harvest location can imply change(s) in secondary metabolite production as their biosynthesis generally depends on the control by both biotic and abiotic factors. Furthermore, the other species of the same genus might very well express different genes coding for proteins acting slightly differently in biosynthetical pathways leading to different compounds or to the same compounds synthetized in different proportions (see below for discussion on diversity). Moreover, if the collected species are not properly reported following the correct taxonomy, the harvest might be even more difficult to reproduce: thereby, a table of correspondence between the names mentioned in this review (and used in the articles of origin) and the accepted plant species names is provided in the supplementary data (**Table S1**).

Another type of limitation is related with the fact that groups performing bioguided discovery of new actives are often confronted with similar problems: (a) the active extracts, once fractionated, do not maintain the level of activity; (b) the whole long and difficult isolation process leads to a molecule that is mundane or known for decades; (c) the final product quantity in the remaining active fraction is too small to hope for a structure elucidation; (d) going back to the location of origin, the same plant population is not found anymore or if the plant is found, the same extraction treatment led to an apparent lack of active fraction(s) on the same target. Most of those events are not discussed in publications, obviously. Therefore, this bioguided approach, existing with small variations––mostly technical––is paved with difficulties that are more than often discouraging at least for an industry or for big programs (Nothias et al., 2018).

As discussed in many instances and particularly well summarized by Atanasov et al. (2015), one of the key starting points is the choice of the pharmacological assay used for the screening. The fact that the targeted disease is obviously more complex than the molecular target it has been simplified to, is universally recognized, but alternative approaches remain elusive or still difficult to set up. For example, the disease could also be simplified by a phenotypic change of a cell originating from a diseased organ using the stem cell differentiation approaches (Bedut et al., 2016). After treating such a model with NP extracts—having potentially hundreds of compounds—reverse pharmacology should allow recognizing the actual target of the NP compound as the lysed cell extract is chromatographed to retain the target proteins (Raut et al., 2017). Nevertheless, cellular phenotypic changes are extremely complex to understand, and this approach has been more than once extremely frustrating despite some successes, outside of the NP recognition area (Nosjean et al., 2000; Graves et al., 2002). Actually, another limitation of this approach is linked with working with complex mixtures in NP discovery as sometimes several compounds could contribute synergistically to the global action on a disease: like for example the compounds (at least three of them) derived from leaves of *Salvia miltiorrhiza* (red sage) that are reported to act at many different levels of liver fibrosis (Ma et al., 2019). This kind of observation would strongly argue against the strategy described in here aiming at the discovery of the main pure drug-like compound in a plant extract. In summary, such approaches (phenotype screening and reverse pharmacology) would require disease models that are far from being the current available systems.

#### Limitations Related to the Need of Market-Compatible Amounts

In the domain of drug production, a fact is that around 60,000 tons per year of salicylic acid are produced synthetically worldwide (https://ihsmarkit.com/products/chemical-technology-pep-reviews-salicylic-acid-2003.html), and this figure translates to 80 billions of pills. That is to say, that in order to benefit patients, critical amounts of a potential drug need to be considered, and the access to the identified NP as well as its availability are critical. For example, at the start of the Taxol™ story, the compound was isolated from *Taxus brevifolia* bark. Ten kilograms of bark was necessary to obtain 2 g of the pure compound needed for the treatment of one patient. For the clinical study, 12,000 trees were cut down to obtain the 2 kg necessary for the studies leading to the approval of the compound. In fact, the antineoplastic Taxotere™ is now produced by hemi-synthesis from the precursor 10-deacetyl-baccatine III, which is isolated from leaves of *Taxus baccata*. In other words, an alternative had to be found to the initial procedure taking a too heavy toll on trees of the initial species. Another interesting figure dealing this time with herbals is the annual demand of ginseng roots around 50,000 tons (Mathur et al., 1999). This kind of figures embodies a major limitation to new drugs originating from NP that is sometimes disregarded by research laboratories: there is a real challenge for the use of NP in reaching a tonnage compatible with the drug market.

Another reason for which NP based projects might be challenged for consideration by the industry is the small amount in which natural compounds generally accumulate in plant tissues. Indeed, structural characterization as well as biological testing can be made more difficult. Nevertheless, many progresses have been done in this area: in relation with structural characterization, for example, considering the growing sensitivity of the analytical methods and instruments, it will not be long until the barrier of the microgram for the determination of the structure of a compound would be reached. The analytical techniques for structural elucidation of an unknown compound are based on a mixture of spectroscopy (infrared, UV, Raman), mass spectrometry, and nuclear magnetic resonance (proton and C^13^ NMR). Nice examples of such task are described in various publications (Baker et al., 1990; El-Elimat et al., 2013) while difficulties are also discussed by Amagata (2010). Advances in NMR techniques were reviewed (Breton and Reynolds, 2013; Harvey et al., 2015; Gomes et al., 2018), and several examples of compounds for which structures were solved with mass of pure compounds lower than 100 µg were reported. In fact, the situation could be different from one plant to another or from one organ/tissue to another, even in a single plant species. Therefore, even if we can identify the many compounds in each sample while being able to establish their structure, it would be more straightforward to consider the feasibility of the systematic compendium of all the plant chemical components.

A careful understanding of the metabolic pathways of the tissues of the plant species from which the product has been isolated is also necessary. It is especially the case when hemi-synthesis is to be used to perform the NP’s industrial production. Instead of using a vital organ of the plant in which the compound is in fair concentration, one tries to find a precursor of the product in a renewable part of the plant, like the leaves. For the hemi-synthesis of Taxol™, 30 km^2^ of *T. brevifolia* fields in the Yunan province (China) consists in the main source of precursor for (http://www.yewcare.com/index.php cited in Malik et al., 2011). Certainly, the use of a renewable source of the natural product is desirable, and often enough that such sustainable and profitable solutions are found, as proven by the 40% of the available drugs in the Pharmacopeia that are from natural origin (Cragg et al., 1997; Newman et al., 2003; Newman and Cragg, 2007; Da Silva and Meijer, 2012; Newman and Cragg, 2012; Newman and Cragg, 2016). Many examples of drugs from natural origin exist, one being found in the still common use of plant sapogenins, alkaloids, or sterols for the production of steroids for human drugs (sex hormones, corticosteroids, contraceptive drugs, *etc*.). Other examples involving hemi-syntheses modifying the NP can be given: quinine (at least 100 tons per year), camptothecin (Asano et al., 2009), cocaine, camphor, vitamin B12, *etc*. Interestingly, at the other end of the spectrum, extremely simple molecules also of NP origin like salicylic acid for pain (J. B. Jin et al., 2017) or metformin for diabetes (Bailey, 2017) see their production reach industrial scales by total syntheses using standard organic chemistry.

### The Need for Easy Access to Chemically Diverse Compounds

Testing as many compounds as possible with chemically diverse structures is important in order to have better chances to discover new drugs (Firn and Jones, 2003). Indeed, the affinity of a drug to a target is the result of shape and electrostatic potential complementarity between the drug and the binding site (Bauer and Mackey, 2019) as well as binding kinetic related properties such as desolvation and conformation changes upon binding. Ligand flexibility therefore plays an important role in identifying partially fitting chemicals as starting points further optimized by medicinal chemistry.

Screened compounds need to be diverse in shape and electrostatics to match any of the 3,300+ binding site, as listed in the pocketome (Kufareva et al., 2012), as well as diverse in structure to allow thorough optimization. It is noteworthy that the probability of identifying a hit considerably decreases with the increasing complexity of the ligand (Hann et al., 2001). The complexity of a molecule increases with the size and the atom connectivity. As a result, the more complex they are, the greater the number of molecules one should screen. Statistical analyses have been performed on natural products to study their chemical diversity (Firn and Jones, 2003; Hong, 2011), molecular properties (Feher and Schmidt, 2003; Quinn et al., 2008), scaffold diversity (Lee and Schneider, 2001; Grabowski et al., 2008; Yongye et al., 2012), and coverage of the chemical space (Rosén et al., 2009a). Comparisons between NP and other compound collections (Henkel et al., 1999; Lee and Schneider, 2001; Grabowski and Schneider, 2007; Rosén et al., 2009a) have also been performed, showing that NP differ from drugs and synthetic compounds in several aspects. NP are considered complex due to their number of asymmetric centers, their number of Sp3 carbon ratio in rings, and their number of ring junctions. Indeed, NP display in general more chiral centers than drugs, although their number of chiral centers to number to carbon atoms ratio may be lower (Gu et al., 2013; Skinnider et al., 2017). Although there is no clear evidence that this complexity is necessary for their biological activity (Firn and Jones, 2003), it greatly impacts their specificity (Clemons et al., 2011). NP also display a great diversity in their scaffolds (Lee and Schneider, 2001; Grabowski et al., 2008; Yongye et al., 2012). For instance, the GreenPharma database (Do et al., 2015; Gally et al., 2017) contains 55,185 Murcko frameworks (Bemis and Murcko, 1996) for 302,000 natural products (18.3%), whereas NCI and ChEMBL databases contain a little of less NP-derived scaffolds: 13.1 and 13.6% respectively.

**Figure 1:**
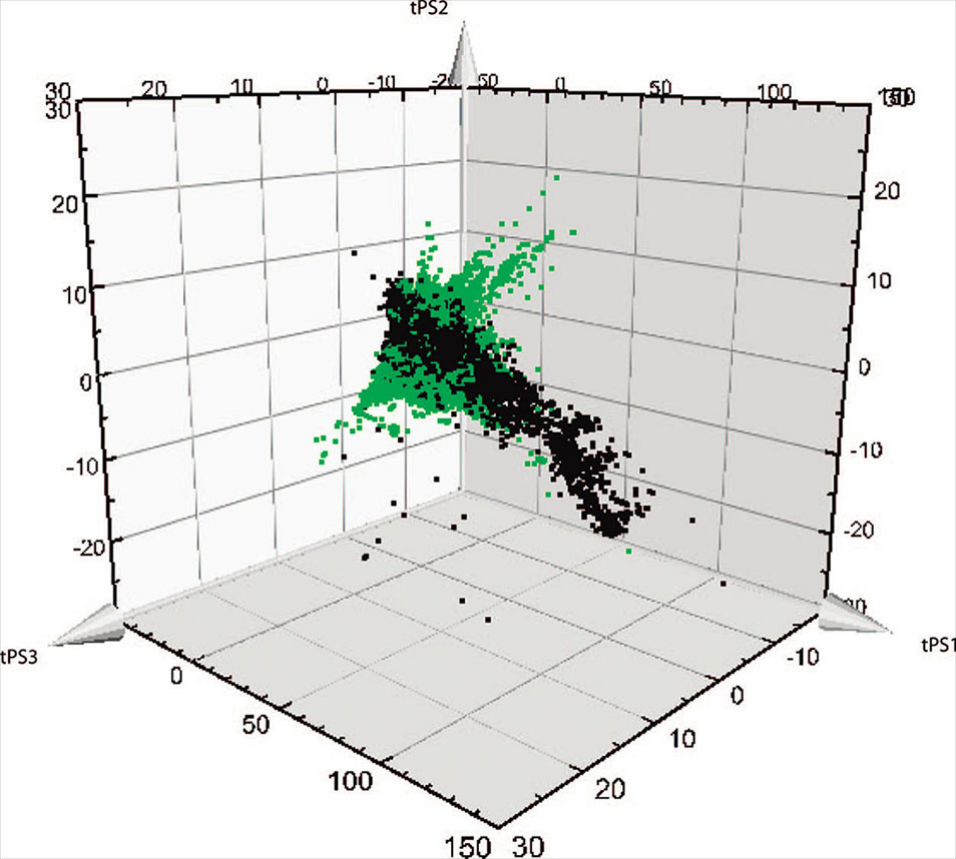
Representation of the divergent diversity between natural and synthetic compounds. (adapted with permission from Rosén et al. (2009a). Copyright 2009 American Chemical Society). Biologically relevant chemical space covered by natural products (in green) or by bioactive medicinal chemistry compounds from the database WOMBAT (in black) in the first three principal components.

An interesting representation of the divergent diversity between bioactive NP and synthetic compounds can be seen in **Figure 1**. This graph reported by Rosén et al. (2009a) shows, on a small sample of compounds (126,140 natural compounds *versus* 178,210 medicinal chemistry-issued compounds), the difference in repartition between these two populations after a principal component analysis. NP are also considered biologically diverse compounds that can hit a vast diversity of biological targets (Hong, 2011). Most of them have a single known target, with a mean of 2.66 targets and only few highly promiscuous compounds. However, biological activity is reported for only 2% of the NP (Gu et al., 2013). An extended study using docking experiments of NP in 332 targets showed a mean of 2.14 targets per natural products, while in comparison, drugs interact in average with 3–6 targets, and 50% of all drugs might exhibit activity against more than five targets (Mestres et al., 2008; Jalencas and Mestres, 2013; Hu et al., 2014).

For all these reasons, there is a strong need for an easy and organized access to chemically diverse structures in the form of libraries of compounds. It is necessary to keep a good balance between diversity, defined as the mean of compound’s pairwise dissimilarity and structural redundancy to ensure greater diversity of hits at an overall hit rate in the range of 0.5 to 2% and 20–40% within an active chemical series. In addition, these compound libraries are required to be constantly enriched with compounds filling the gaps in the molecular space. Interestingly, Harvey et al. (2015) stated: “diversity within biologically relevant chemical space is more important than library size”, this biologically relevant chemical space being defined by protein-binding sites for potential ligands. Another point emphasized by these authors is that while the commercially available chemical space is wider than the explored natural product universe—the first one is evaluated to 5.10^9^ compounds, while the second only represents 3.10^5^ compounds (Banerjee et al., 2015)—NP are intrinsically more diverse. A major component of this comparison is that NP are more complex, from a chemical point of view, leading to greater shape diversity. However, NP might not be the best compounds to screen in HTS campaigns due to their complexity (“best” in a drug-discovery perspective). Indeed, their probability of being active on a random target is lower, and their chemical tractability is far from optimal. Although NP decently occupy the biologically relevant chemical space, and 80% meet the criteria to be considered as drug like compounds (Harvey et al., 2015), their complexity might be the bottleneck during the optimization phase of the lead compounds.

Thus, acquiring as many diverse compounds as possible is necessary in drug discovery programs, and the exploration of natural existing chemical diversity would be a must. Nevertheless, not only the design of those libraries, but also the design of the assays and the understanding of the pathways that are responsible for the targeted pathologies need to be relevant and well understood. In summary, the main idea is that if compounds as chemically diverse as possible are necessary to feed drug discovery programs, then a great deal of efforts should still be invested in the exploration of natural chemical diversity because, in this particular context, natural chemistry is more advanced than organic chemistry. After this introduction on the background of NP in the area of drug discovery, we aim at discussing and showing the extent of natural chemical diversity (*Natural Chemical Diversity*) as well as the strategies of having the potential to embrace this diversity (*Different Strategies to Benefit From This Diversity*) based on an extensive survey of recent publications. A preliminary survey on the main origins of recently reported natural compound structures’ literature was performed by taking a complete volume from a journal that is considered as a gold standard in NP (J. Natural Products, 2018, vol 81). Then three different tables mentioning the species of origin and the type of compounds newly reported in 2017 and 2018 in several journals selected for their specialization in the area (like for example Journal of natural products, Planta medica, Tetrahedron Letters or Natural products communications) were built upon this sampling. Finally, *Different Strategies to Benefit From This Diversity* offers some strategic and technical proposals based on our interpretations of the trends of the field supported by recent *ad hoc* literature.

## Natural Chemical Diversity

The chemical diversity of NP is known to be extremely wide, and it can be divided in several types representing a challenge for the chemist and the biologist.

### Diversity of Origins in Natural Compounds

Diversity can be measured and quantified by many methods some of which have been used for decades to qualify the product library diversity (Langer et al., 2009). Our first survey showed the main origins of the molecules reported as natural compounds in 2018: they were classified between those coming from animals (2.6%), from fungi and lichens (9.3%), from microorganisms (12.9%), from marine organisms in the broadest sense of the term (13.2%), and from plants (44.1%), while the papers dealing with partial or total syntheses of natural compounds (19) and about 20 others describing various methodologies of analyses were put aside. As shown, in 2018 plants were still the main source of “new” natural compounds.

Then, by sampling the recent *ad hoc* literature, three different tables were constructed gathering the origin of new compounds reported in 2017 and 2018 in several journals selected for their specialization in the area. Although plants were our main focus, we thought it might be useful to also consider other sources of diversity from other living kingdoms as they also consist of very interesting sources to explore and because some of them are somehow related to plants: it is especially the case for compounds which functions in plants are related with defense (Bednarek and Osbourn, 2009) and other ecological interactions as compounds “optimized by evolution” according to Gunatilaka in its review on natural products from plant-associated microorganisms (Gunatilaka, 2006). Among the 240 papers that were reviewed, a handful described compounds isolated from venoms and toxins originating from marine organisms and animals (insects, snakes, and alike). Then, a few other papers described compounds coming from microorganisms as can be seen in **Table 2**. Microorganisms, such as bacteria and yeasts, were traditionally important sources of antibiotics and are still a source of new peptides (Xue et al., 2018), but here compounds other than peptides can also be found that are close to secondary metabolites naturally synthesized in those cells. New peptides are more often described in journals specialized in peptide chemistry and pharmacology, even if their source is a living organism. **Table 3** gives examples of compounds isolated from lichen, fungi, and sponges. The chemical diversity of these compounds is interesting but, as of today, growing lichen or sponges is not so much reported and might turn out to be difficult on a larger scale. On the other hand, secondary metabolites synthetized by microorganisms growing in extreme conditions might also be difficult to obtain in large quantities. It would be therefore interesting to dig deeper in this area, as it has been done elsewhere for other purposes: biotechnological (Kruger et al., 2018; Mokashe et al., 2018), industrial (Sarmiento et al., 2015), pharmaceutical (Irwin, 2010; Karker et al., 2016; Patel, 2016; Oliveira et al., 2018), or ecological (Casillo et al., 2018; Orellana et al., 2018). Finally, **Table 4** gives many examples of compounds isolated from different plant species and different tissues from these species.

**Table 2:** Some examples of compounds isolated from microorganisms.

Species	Compound	Reference
*Hypoxylon. Monticulosum*	Sporothriolide derivatives	(Leman-Loubiere et al., 2017)
*Malbranchea flavorosea*	X (*)	(Verastegui-Omana et al., 2017)
*Micromonospora. Harpali*	Spirotetronate	(Gui et al., 2017)
*Moorea. Producens*	Cryptomaldamide	(Kinnel et al., 2017)
*Okeania* sp.	Kohamamides	(Iwasaki et al., 2017)
*Paecilomyces* sp.	β-Resorcylic Acid Lactones	(Xu L. et al., 2017)
*Palicourea sessilis*	Monoterpene indole alkaloids	(Klein-Junior et al., 2017)
*Penicillium commune*	Cyclopiane diterpenes	(Niu et al., 2018)
*Plasmodium falciparum*	Octaminomycins	(Jang et al., 2017)
*Pseudonocardia carboxydivorans*	Branimycins	(Brana et al., 2017)
*Pseudovibrio denitrificans*	Alkaloids	(Nicacio et al., 2017)
*Rhytismataceae* sp.	X (*)	(McMullin et al., 2017)
*Serratia plymuthica*	Plymuthipyranones	(Bjerketorp et al., 2017)
*Streptomyces misionensis*	Streptenols	(Tarazona et al., 2017)
*Streptomyces* sp.	Spoxazomicin D	(Shaaban et al., 2017)
*Streptomyces* sp.	Dibohemamines	(Jiang et al., 2017)
*Symploca* sp.	Caracolamide	(Naman et al., 2017)
*Thalassospira profundimaris*	Thalassosamide	(Zhang et al., 2017)
*Thermoactinomyces. Vulgaris*	Thermoactinoamide A	(Teta et al., 2017)
*Trichoderma sp.*	Neomacrophorin X	(Kusakabe et al., 2017)

(*) those papers described many different compounds in those microorganisms.

**Table 3:** Some examples of compounds isolated from fungi, sponges and lichens.

Species	Compound(s) found in Fungus, Sponge, Lichen	Reference
*Acanthostrongylophora* sp.	Manzamine Alkaloids	(Kim et al., 2017)
*Amphimedon* sp.	Zamamidine D	(Kubota et al., 2017)
*Annulohypoxylon truncatum*	Isochromans	(Li et al., 2017f)
*Antrodia cinnamomea*	Antroquinonol derivatives	(Chen et al., 2017)
*Aspergillus ochraceopetaliformis*	Ochracenes	(Wang et al., 2017b)
*Aspergillus* sp.	Protulactone A	(Markovic et al., 2017)
*Auxarthron pseudauxarthron*	Phenalenones	(Li et al., 2017)
*Botrysphaeria laricina*	Botrysphones	(Zhang et al., 2017)
*Chondrostereum* sp.	Hirsutane Sesquiterpenes	(Hsiao et al., 2017)
*Clathria gombawuiensis*	Gombasterols	(Woo et al., 2017)
*Cochliobolus australiensis*	Chloromonilinic Acids	(Masi et al., 2017)
*Delitschia. Confertaspora*	Fimetarone	(Jayanetti et al., 2017)
*Diaporthe toxica*	Phomopsin A	(Schloss et al., 2017)
*Emericella* sp.	Emervaridones	(He et al., 2017)
*Ganoderma applanatum*	Spiroapplanatumines	(Luo et al., 2017)
*Glomerella cingulata*	Sesquiterpenes	(Liu et al., 2017a)
*Hyrtios* sp.	Meroterpenoids	(Wang et al., 2017c)
*Ircinia oros*	Furanosesterterpenoids	(Chianese et al., 2017)
*Lamellodysidea herbácea*	Lamellodysidines	(Torii et al., 2017)
*Lobaria orientalis*	Lobarientalones	(Nguyen et al., 2017)
*Monanchora unguiculata*	Unguiculin A	(Campos et al., 2017)
*Montagnulaceae* sp.	Montagnuphilones	(Luo et al., 2017)
*Oscarella stillans*	Oscarellin	(Kwon et al., 2017)
*Paecilomyces gunnii*	Gunnilactams	(Zheng et al., 2017)
*Paraconiothyrium* sp.	Versiol derivatives	(Cho et al., 2017)
*Penicillium concentricum*	Halogenated compounds	(Ali et al., 2017)
*Penicillium janthinellum*	Penicilones	(Chen et al., 2017)
*Penicillium janthinellum*	Penisulfuranols	(Zhu et al., 2017)
*Phaeoacremonium* sp.	Isoaigialones	(Silva et al., 2017)
*Preussia similis*	Preussilides	(Noumeur et al., 2017)
*Preussia. Minimoides*	Minimoidiones	(Rangel-Grimaldo et al., 2017)
*Psammocinia* sp.	Sulawesins	(Afifi et al., 2017)
*Spongia ceylonensis*	Ceylonins	(El-Desoky et al., 2017)
*Spongia pertusa*	Sesquiterpene quinones	(Li et al., 2017a)
*Stachybiotrys chartarum*	Stachybotrysins	(Zhao et al., 2017)
*Stereocaulon paschale*	Dibenzofuranes	(Carpentier et al., 2017)
*Talaromyces aculeatus*	Azaphilone derivatives	(Ren et al., 2017)
*Talaromyces islandicus*	Hydroanthraquinones	(Li et al., 2017)
*Talaromyces* sp.	Talarazines	(Kalansuriya et al., 2017)
*Theonella swinhoei*	Cyclotheonellazoles	(Issac et al., 2017)
*Topsentia* sp.	Tulongicin	(Liu et al., 2017)
*Trichoderma gamsii*	Trichoderpyorone	(Chen et al., 2017)
*Xylaria nigripes*	X (*)	(Chang et al., 2017)

Compounds originating from fungus in black, from sponge in red, and from lichen in green; (*) this paper described many different compounds in this fungus.

**Table 4:** Compounds from plant parts.

Species	Plant leaf	Whole aerial plant and stems	Plant rhizomes and roots	Plant fruit and flowers	Plant bark, wood and seeds	Ref
*Acacia ligulata*				Ligulatasides		(Jaeger et al., 2017)
*Acorus tatarinowii*			Asarone derivatives			(Gao et al., 2017)
*Aconitum apetalum*			Apetaldines			(Zhang et al., 2017)
*Allanblackia floribunda*					Xanthones	(Mountessou et al., 2018)
*Alnus viridis*					**Hydroxyalphitolic acid derivatives**	(Novakovic et al., 2017)
*Althaea officinalis*			X (*)			(Sendker et al., 2017)
*Amorpha fruticosa*				Amorphispironones		(Muharini et al., 2017)
*Anabasis articulata*		nor-Triterpenoidal Saponins				(Salah El Dine et al., 2019)
*Ancistrocladus ileboensis*	Jozilebomines					(Li et al., 2017c)
*Ancistrocladus ileboensis*	Dioncophyllines					(Li et al., 2017d)
*Anigozanthos* sp.				X (*)		(Hendra and Keller, 2017)
*Anthemis nobilis*				Sesquiterpene lactones		(Mieri et al., 2017)
*Aphanamixis polystachya*	Limonoids					(Camero et al., 2018)
*Aquilaria malaccensis*					**Aquilanols**	(Ma et al., 2017)
*Aquilaria malaccensis*					Phorbol esters	(Wagh et al., 2017)
*Aquilaria sinensis*					chromen-4-one	(Wang S.-L. et al., 2018)
*Aristolochia orbicularis*			Aristoloxazines			(Rios et al., 2017)
*Artocarpus rigida*		Artocarmins				(Nguyen et al., 2017)
*Atalantia monophylla*				Atalantums		(Sribuhom et al., 2017)
*Azadirachta indica*	Tamarixetin glucopyranoside					(Yadav et al., 2017)
*Baeckea frutescens*	Phloroglucinol meroterpenoids	**Phloroglucinol meroterpenoids**				(Qin et al., 2018)
*Baeckea frutescens*		**Frutescones**				(Hou et al., 2017)
*Balthasaria mannii*				Pyran-2-ones derivatives		(Ahoua et al., 2018)
*Belamcanda chinensis*			Belamcandanes			(Ahoua et al., 2018; Ni et al., 2018)
*Berchemia berchemiifolia*				Berchemiosides		(Kang et al., 2017b)
*Betula pendula*					Betulin derivatives	(Tsepaeva et al., 2017)
*Betula pubescens*					Suberin fatty acids	(Heinamaki et al., 2017)
*Boesenbergia pandurata*			Cyclohexene chalcones			(Nguyen et al., 2017b)
*Bougainvillea spectabilis*					**Bougainvinones**	(Do et al., 2018)
*Bowdichia virgilioides*					Sucupiranines	(Endo et al., 2017)
*Buddleja asiatica*		Iridoid glycosides				(Hien et al., 2018)
*Bupleurum fruticosum*	Phenylpropenoids					(Fois et al., 2017)
*Calotropis gigantea*				Uscharin		(Yoneyama et al., 2017)
*Camellia crapnelliana*	Camellianols	Camellianols				(Xiong et al., 2017)
*Carpesium cernuum*		**Carpescernolides**				(Yan et al., 2018))
*Caryopteris Nepetaefolia*		Nepetaefolins				(Zhang et al., 2017)
*Catalpa ovata*					Catalpol derivatives	(Kil et al., 2017)
*Celastrus subspicata*	Celastrofurans					(Wibowo et al., 2017)
*Cephalotaxus fortunei*	Cephalotaxus troponoids					(Zhao et al., 2017)
*Cephalotaxus sinensis*	Cephanolides	Cephanolides				(Fan et al., 2017)
*Ceratodon purpureus*		Biflavonoids				(Waterman et al., 2017)
*Chaenomeles sinensis*		Sinenic acid A analogues				(Kim et al., 2017b)
*Chrysanthemum morifolium*				Caffeoylquinic acid		(Yang et al., 2017)
*Chrysanthemum indicum*				Chrysanthemumins		(Liu et al., 2017)
*Cimicifuga dahurica*			**Cimiricaside B**			(Thao et al., 2017)
*Cinnamomum cassia*		Caffeic acid phenethyl ester				(Shin et al., 2017)
*Clausena anisumolens*	Anisucoumaramide					(Wang et al., 2017)
*Cleistochlamys kirkii*	Cleistodienol derivatives					(Nyandoro et al., 2017a)
*Codonopsis pilosula*			Piluloside			(Zheng et al., 2018)
*Coleonema album*	Coumarins					(Lima et al., 2017)
*Cornus officinalis*				Cornusides		(Ye et al., 2017)
*Curcuma aromatica*			Sesquiterpenoids			(Dong et al., 2017)
*Curcuma longa*		Cyclocurcumin				(Kim et al., 2017)
*Cyclopia intermedia*		Polyphenols				(Jack et al., 2018)
*Cynomorium songaricum*		X (*)				(Ma et al., 2018)
*Dasymaschalon echinatum*		Aristolactams				(Bunteang et al., 2018)
*Eremophila longifolia*	Nerol cinnamates					(Galappathie et al., 2017; Nyandoro et al., 2017b))
*Erythrina schliebenii*					Isoflavones	(Nyandoro et al., 2017b)
*Euphorbia fischeriana*			ent-Abietane derivatives			(Wang et al., 2017)
*Euphorbia gaditana*		Gaditanone				(Flores-Giubi et al., 2017)
*Euphorbia kansui*			Ingenane Diterpenoids			(Zhang et al., 2018)
*Euphorbia pithyusa*		Premyrsinane				(Esposito et al., 2017)
*Euphorbia semiperfoliata*		Jatrophane esters				(Nothias et al., 2017)
*Euphorbia soongarica*		Sooneuphoramine				(Gao and Aisa, 2017)
*Euphorbia taurinensis*	Diterpenes					(Rédei et al., 2018)
*Euphorbia ebracteolata*			Abietane derivatives			(Wei et al., 2017)
*Excoecaria agallocha*		nor-Oleanane triterpenes				(Mo et al., 2018)
*Ficus fistulosa*	**Phenanthroindolizidine Alkaloid**					(Al-Khdhairawi et al., 2017)
*Fissistigma latifolium*					Ecarlottones	(Geny et al., 2017)
*Flindersia pimenteliana*	Pimentelamines					(Robertson et al., 2017)
*Forsythia suspensa*				X (*)		(Kuo et al., 2017)
*Garcinia propinqua*					Xanthones	(Sriyatep et al., 2017)
*Gardenia ternifolia*	Gardenifolins					(Tshitenge et al., 2017)
*Gloriosa superba*					Gloriodiside	(Zarev et al., 2017)
*Glycyrrhiza glabra*			**Glycybridins**			(Li et al., 2017)
*Hoya kerrii*		Epoxypregnane				(Seeka et al., 2017)
*Humulus japonicus*	Humulusides					(Yang et al., 2018)
*Humulus lupulus*				Xanthohumol		(Liu et al., 2017)
*Humulus lupulus*				α-acid derivatives		(Li et al., 2017b)
*Hunteria zeylanica*	Hunterizeylines					(Bao et al., 2017)
*Hypericum henryi*		**Hyperhenones**				(Duan et al., 2018)
*Hypericum perforatum*	Acylphloglucinols					(Guo et al., 2017)
*Hyptis brevipes*		Brevipolides				(Suarez-Ortiz et al., 2017)
*Impatiens balsamina*				Balsamisides		(Kim et al., 2017a)
*Indigofera stachyodes*			Stachyodin A			(Zhang et al., 2018)
*Iris tectorum*	Polycycloiridals					(Zhang et al., 2017)
*Isodon pharicus*		Pharicins				(Hu et al., 2018)
*Isodon scoparius*		ent-kaurane derivatives				(Jiang et al., 2017)
*Jatropha dioica*			Riolozane derivatives			(Melchor-Martinez et al., 2017)
*Juglans regia*				Azacyclo-indoles		(Li et al., 2017)
*Justicia gendarussa*		**Patentiflorin A**	**Patentiflorin A**			(Zhang et al., 2017a)
*Kopsia officinalis*	Rhazinilam					(Zeng et al., 2017)
*Laetia corymbulosa*					**Corymbulosins**	(Suzuki et al., 2017)
*Lepidozia reptans*	Terpenoids					(Li S. et al., 2018)
*Leplaea mayombensis*			Leplaeric acid derivatives			(Sidjui et al., 2017)
*Ligularia fischeri*		Diterpenoids				(Gobu et al., 2017)
*Liquidambar formosana*	**Isorugosins**					(Shimozu et al., 2017)
*Lithocarpus litseifolius*	**Triterpenoids**	**Triterpenoids**				(Cheng et al., 2018)
*Litsea cubeba*		Glycosides				(Wang et al., 2017)
*Litsea cubeba*		Isoquinoline alkaloids	Isoquinoline alkaloids			(Yang et al., 2018)
*Macaranga tanarius*				Schweinfurthins		(Peresse et al., 2017)
*Mammea harmandii*					Xanthones	(Liangsakul et al., 2018)
*Millettia oblata*	Rotenoids					(Deyou et al., 2017)
*Momordica balsamina*				Triterpenoids		(Ramalhete et al., 2018)
*Momordica charantia*				Cucurbitanes		(Tuan et al., 2017)
*Nauclea orientalis*			Indole alkaloids			(Surapinit et al., 2018)
*Ongokea gore*					Hydroxy diynes	(Ntumba et al., 2018)
*Panax ginseng*				Malonylginsenosides		(Qiu et al., 2017)
*Paramignya trimera*		Quinoliniumolate				(Nguyen et al., 2017a)
*Paulownia tomentosa*				Tomentins		(Ryu et al., 2017)
*Peganum harmala*				Peganine derivatives		(Wang et al., 2017)
*Pentalinon andrieuxii*			Pentalinonsterol			(Oghumu et al., 2017)
*Pentarhizidium orientale*			**Matteuorienates**			(Huh et al., 2017)
*Peperomia obtusifolia*	Orsellinic acid derivatives	Orsellinic acid derivatives	Orsellinic acid derivatives			(Batista et al., 2017)
*Perovskia abrotanoides*		Diterpenes				(Tabefam et al., 2018)
*Peucedanum japonicum*			**Khellactone Esters**			((Hong and Kim, 2017)
*Phoradendron vernicosum*		Lupane derivatives				(Valencia-Chan et al., 2017)
*Phyllanthus acidus*	t-Muurolol					(Pérez-Colmenares et al., 2018)
*Phyllanthus flexuosus*			Purine derivative			(Zhu et al., 2018)
*Physalis peruviana*		Whithanolides				(Xu Y.-M et al., 2017)
*Plectranthus africanus*		**Abietane-derivatives**				(Nzogong et al., 2018)
*Podocarpus nagi*					Nagilactone derivatives	(Feng et al., 2017)
*Polygala flavescens*		X (*)				(Leo et al., 2017)
*Pongamia pinnata*					Furanoflavones	(Sharma et al., 2018)
*Poupartia borbonica*	Poupartones					(Ledoux et al., 2017))
*Prangos haussknechtii*		Prenylated coumarins				(Dissanayake et al., 2017)
*Pulicaria undulata*		**Asteriscunolides**				(Boumaraf et al., 2017)
*Radula sumatrana*		**Rasumatranins**				(Wang et al., 2017)
*Raphanus sativus*	Monoglyceride					(Ogihara et al., 2017)
*Rhodocodon campanulatus*	Bufadienolides					(Schwikkard et al., 2017)
*Rhodomyrtus tomentosa*	Tomentodiones					(Zhang et al., 2017)
*Salvia chamaedryoides*	Diterpenoids					(Bisio et al., 2017)
*Salvia circinata*		Neoclerodane glucosides				(Flores-Bocanegra et al., 2017)
*Salvia miltiorrhiza*	**Salvianans**	**Salvianans**				(Zhang et al., 2017)
*Salvia plebeia*		epi-Eudebeiolides				(Jang et al., 2017)
*Salvia polystachya*		Polystachynes				(Bautista et al., 2017)
*Sambucus williamsii*		Iridoid glycosides				(Suh et al., 2017)
*Saxifraga spinulosa*		Galloyl glycosides				(Badral et al., 2017)
*Schisandra bicolor*				Shibitubins		(Liu et al., 2017b)
*Scutellaria barbata*		neo-Clerosane diterpenoids				(Yang et al., 2018)
*Selaginella pulvinata*		Diselaginellin				(Cao et al., 2017)
*Siegesbeckia pubescens*	ent-Strobane					(Wang et al., 2017a)
*Sinningia reitzii*			Dunnione derivatives			(Soares et al., 2017)
*Siraitia grosvenorii*				Cucurbitane		(Niu et al., 2017)
*Streblus asper*					**Kamaloside derivatives**	(Ren et al., 2017)
*Strychnos icaja*			Strychnogucine B			(Beaufay et al., 2018)
*Tabernaemontana bufalina*		Bisendol alkaloids				(Zhou et al., 2018)
*Taxus wallichiana*					**Wallitaxanes**	(Dang et al., 2017a)
*Taxus wallichiana*			Lignans			(Dang et al., 2017b)
*Teucrium yemense*		Neoclerodane Diterpenoids				(Nur-E-Alam et al., 2017)
*Thalictrum cultratum*			Aporphinoid alkaloids			(Li et al., 2017)
*Tinospora sagittata*			Bistinospinosides			(Li et al., 2017e)
*Tinospora sinensis*		Tinosinenosides				(Jiang et al., 2017)
*Trichospira verticillata*	Sesquiterpenoid Lactones					(Du et al., 2017)
*Typhonium giganteum*			Cerebrosides			(Jin et al., 2017)
*Uncaria rhynchophylla*		**Dimeric isoechinulin**				(Geng et al., 2017)
*Uvaria alba*	Albanols					(Macabeo et al., 2017)
*Vaccinium ashei*				Anthocyanins		(Huang et al., 2018))
*Vallaris glabra*	Cardenolide glycosides					(Kruakaew et al., 2017)
*Verbesina lanata*				Eudesmane sesquiterpenes		(Ramseyer et al., 2017)
*Vernonia cinerea*				Hirsutinolide analogues		(Kuo et al., 2018)
*Vitex trifolia*	Vitepyrroloids					(Luo et al., 2017)
*Xylocarpus granatum*					Xylomexicanins	(Wu et al., 2017)
*Xylocarpus rumphii*					Xylorumphiins	(Waratchareeyakul et al., 2017)
*Zephyranthes carinata*		Plicamine derivatives				(Zhan et al., 2017)
*Zizyphus jujuba*					Epicatechinoceanothic acids	(Kang et al., 2017a)

Compound names in bold characters are those that are exemplified in **Figure 1**. Compounds in green were extracted from stems or seeds; in red from roots; in blue from flowers or wood; and in black from leaves, hole aerial part, rhizomes, fruit or bark; (*) those papers described many different compounds in those plant parts. Lines overlaid in gray exemplify similar genuses expressing different compounds.

As plants are one of the major sources of “new” compounds, the following sections will deal with their diversity.

### Diversity of Plant Species

As mentioned, the compounds reported from plants (**Table 4** of our survey) originated from 165 different plant species, from trees to ornamental plants and harvested in very diverse areas from Australia to Antarctica.

**Figure 2:**
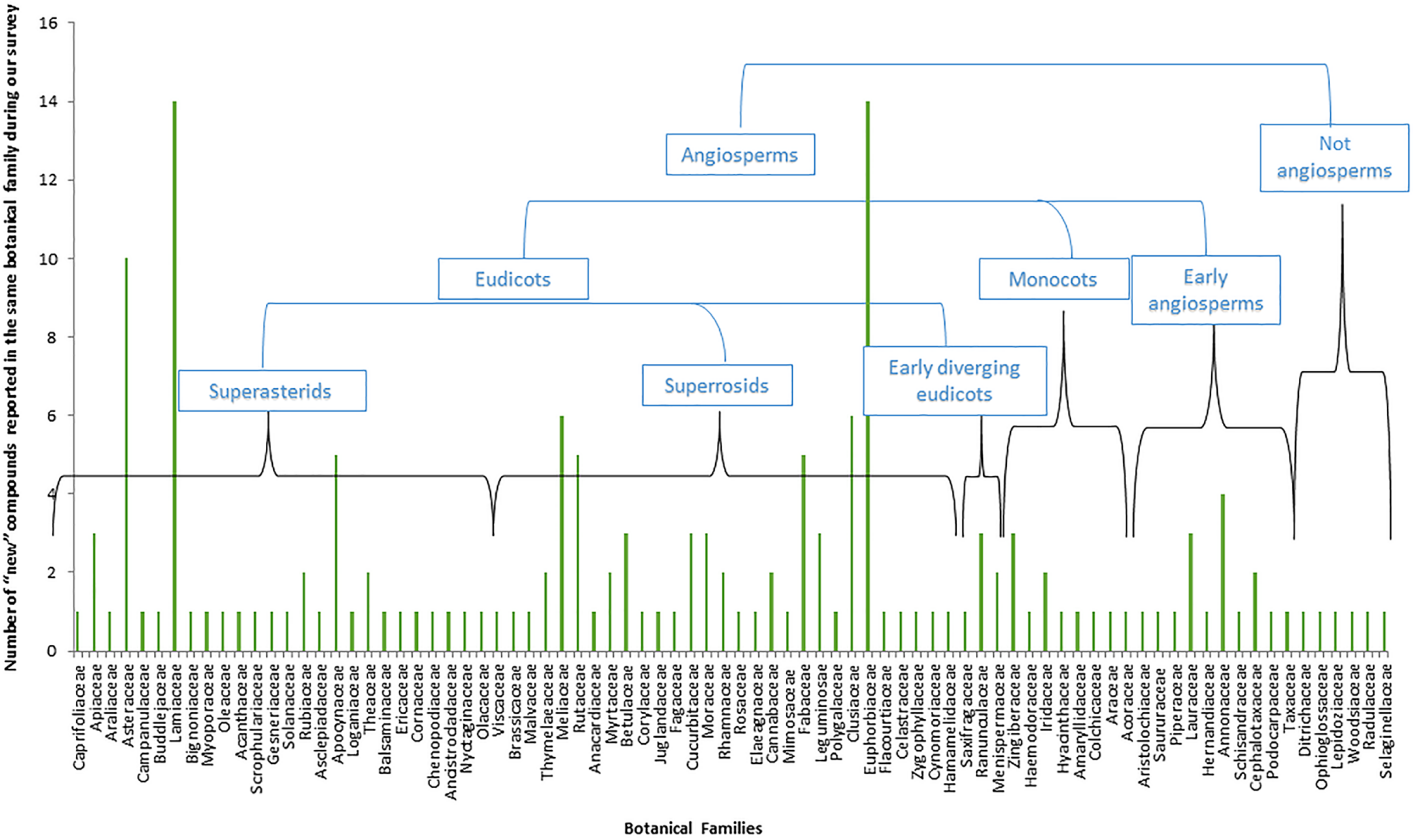
Representation of the relative weight of the different phylogenetic groups in the number of “new” compounds reported based on our survey (**Table 4**).

In fact, a total of 146 different genera were represented from 82 different botanical families, only six of which are not Angiospems (two ferns, two liverworts, a moss, and a spikemoss). Most of them, around 93%, are thus from the flowering plant group (**Figure 2**). Furthermore, it is interesting to observe that the remaining 76 families from which these new compounds were reported represent a small part of the total 416 botanical families defined in the last 2016 update of the Angiosperms Phylogeny Group (The Angiosperm Phylogeny Group et al., 2016). As illustrated, most of the new compounds of our survey were isolated from the superasterids and superrosids, major clades from the eudicots. Indeed, this small survey illustrates how a large proportion of plant species still remains underexplored, not only in the Angiosperms but between all taxa of Plant kingdom.

Furthermore, it is important to consider that new species of plant are discovered quite often: for example, 2,034 new plant species were recorded in 2014, including at least one tree species (Mancuso, 2018). So far, 310,000 plant species have already been described, among which authors estimate that only 6% have been investigated pharmacologically and 15% phytochemically (Atanasov et al., 2015). It clearly indicates that even more compounds remain to be found in plants.

It is also interesting to observe that reviews such as Solyomváry et al. (2017) teach us that plants from different taxa can also synthesize identical although rather complex compounds.

Gene duplication and neo-functionalization leading to the extension of the existing metabolic pathways are both part of the mechanisms that have been identified in plants as responsible for diversification of secondary metabolites together with the influence of ecological factors: for example, it has been suggested that, from a small group of precursors, plants would synthetize a full range of highly diverse compounds rapidly changing that are “screened” for their biological activity afterwards as mechanisms of adaptation that would help plants cope better with biotic and abiotic pressures (Moore et al., 2014). This ecological understanding of plant secondary metabolite diversification also contributes to the anticipation that the more plants species are described, the more diversity is to be found in the end-products of these pathways along with possibly valuable “new” compounds. A recent interesting study (Henz Ryen and Backlund, 2019) discussed the chemical Angiosperms’ diversity among natural products and some of these evolutionary mechanisms leading to diversification of chemical structures in function of the group of secondary metabolites (flavonoids, tropane alkaloids, sesquiterpene lactones, and betalains). They use the ChemGPS-NP developed previously by their group (Rosén et al., 2009b) as tool to localize compounds in the chemical property space, “measuring” this way the chemical diversity.

In other words, the preservation of ecological niches and plant biodiversity will also serve our interest in terms of chemical diversity for possible applications. And, interestingly, at higher trophic levels, it will also contribute to biodiversity (Schuman et al., 2016), creating in turn other biotic pressures on plants that may adapt by synthetizing other compounds!

### Chemical Diversity in Plants Related to Space and Time

**Figure 3:**
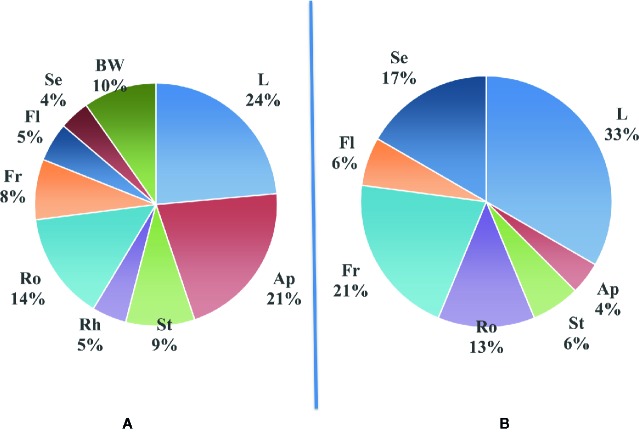
Distribution of newly reported compounds according to the part of plants from which they were extracted. **(A)**: Proportion of new compounds reported in different parts from the plant (from our survey); **(B)**: Proportion of active compounds in different plant parts reported in Ross work (Ross, 1999; Ross, 2001). Ap, aerial parts; BW, bark and wood; Fl, flowers; L, leaves; Fr, fruit; Rh, rhizome; Ro, roots; Se, seeds; St, stems.

As plants are multicellular organisms with organs specialized for different functions, it seems logical to think that some biosynthetic pathways could be turned on or off depending on the part of the plant studied and that a certain level of diversity can exist within the plant tissues. The main parts of the plants that are classically separated are roots, twigs (stems), leaves, flowers, fruits, and seeds. Of course, constraints of collection make some of these parts more suitable than others. Our survey in **Table 4** details for each one of the 165 reported plant species the plant part from which it was isolated. **Figure 3A** summarizes the proportion of new compounds reported in different plant parts in our survey. For comparison, **Figure 3B** shows similarly the organs/parts of origin of the active compounds reported from the 49 plants described in Ross work (Ross, 1999; Ross, 2001). It seems that in both cases, most of the studies are done on leaves. On the other hand, it is not clear, on a systematic basis, if what was found in the other organs can be found as well—even in small quantities—in the leaves. Nevertheless, leaves are the most accessible part of any plant, and they are renewable which allows preserving the whole plant. Obviously, it is also valid for fruits or flowers, but their availability may be subjected to seasonality.

Indeed, often enough, a compound is described from a part of a given plant. It seems interesting to emphasize that it does not necessarily mean that it is the main component or a major component of this plant part. It can be accumulated in superior amounts in other tissues of the same plant. Indeed, in plant descriptions such as those in Ross work (Ross, 1999; Ross, 2001), the author described the way plants are used in traditional medicine. The different parts of a given genus can be used alone or in mixtures in preparations ranging from infusion, maceration, decoction, juice, dried powder, or even fresh organs (fruits, leaves) ingested as such. Astonishingly, those recipes were collected from scattered geographic places. This last observation suggests that traditional medicines around the world independently found similar remedies to similar diseases and sometimes with the use of related plant species.

Another interesting source of intraspecific chemical diversity is the environment of the plant cells biosynthesizing the chemical compounds of interest: it is well known that compounds produced by the same plant species can vary in nature or quantity depending on the environment (localization or time of the year) or the part of the plant where it has been extracted from. Moore et al. (2014) pointed out in particular the plant ontogeny, but also genetic and environmental variations as major sources of diversity for plant secondary metabolites. The existence of chemotypes is another example of this intraspecific chemo-diversity well described for example in plants producing essential oils. Factors like moisture, salinity, temperature, or nutrition levels are known to influence the essential oil production (Sangwan et al., 2001), and the genotype could also significantly influence the chemotype as it was shown recently for *Valeriana jatamansi* Jones (He et al., 2018). The biosynthesis of natural products can also differ in function of the different individuals from the same population. It is the case, for example, when these compounds are related to antimicrobial activity as summarized by Bednarek and Osbourn (2009) in their perspective article on chemical diversity linked with plant defense: these compounds can be synthetized constitutively as part of normal plant development—and stored in specialized tissues—or synthetized in response to pathogenic challenges through the activation of the transcription of specific genes of the corresponding biosynthetic pathways.

### Diversity of Chemical Skeletons and Structures

Based on the number of genes, it has been estimated that the plant kingdom contains more than 200,000 different metabolites with values for single species ranging between 5,000 and 15,000 (Trethewey, 2004; Fernie, 2007), values that are significantly greater than those of microorganisms (∼1,500) and animals (∼2,500) (Oksman-Caldentey and Saito, 2005). But it is not only the global absolute value of chemically diverse compounds that is interesting. Such diversity and such dynamicity are indeed a wonderful wealth of chemical structures, source of inspiration for medicinal chemist, once the structure is carefully identified, and the related pharmacological activity is screened. But it can also become a source of complexity for the phytochemist working on the structure or on the structure/activity relation.

Classically, within this chemical diversity in plant secondary metabolites, the nomenclature used by pharmacologists to attempt classifying several families of natural compounds such as polyketides, phenylpropanoids, terpenoids, steroids, or alkaloids is based more on their biogenesis and the pathway they originate from (acetate, shikimate, mevalonate or methylerythritol phosphate pathways) or their combination, than their structure itself. And even within a defined group, the diversity can be impressive: for example, the terpenoid family is suspected to contain at least 50,000 different molecules (Kirby and Keasling, 2009) while at least 12,000 flavonoids have been described (Henz Ryen and Backlund, 2019). Certainly, how “different” these molecules are could be further commented, but as discussed below, apparently minor differences (for example a methyl or a hydroxyl moiety) might dramatically change the molecule’s pharmacological properties.

**Figure 4:**
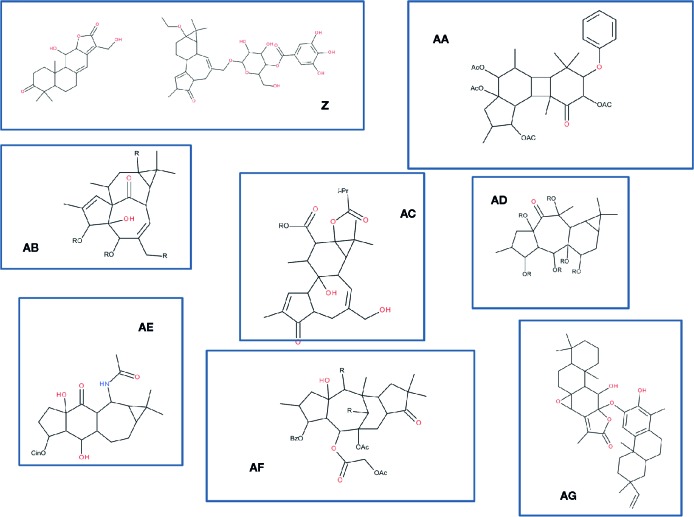
Variations around diterpenoids found in six species of *Euphorbia*. Compounds were drawn from the works on *Euphorbia*. **Z** : ent-abietane-type diterpenoid (**Z**1) and a tiglane (**Z**2) (Wang et al., 2017); **AA**: gaditanone (Flores-Giubi et al., 2017); **AB**: ingenane-type diterpenoid, euphorkan A (Zhang et al., 2018); **AC**: jatrophane-type ester (Nothias et al., 2017); **AD**: dideoxyphorbol ester (Esposito et al., 2017); **AE**: diterpenoid alkaloid sooneuphoramine (Gao and Aisa, 2017); **AF**: jatrophane-type diterpenoid (Rédei et al., 2018); **AG**: abietane-lactone- and nor-rosane-based heterodimeric terpenoids (Wei et al., 2017). Note that the stereospecificity of the compounds was not given, as many different compounds were described in each of those references.

In the data gathered in **Table 4**, as previously commented, it can be observed that even in the same plant species and same organ, different compounds have been characterized like jozilebomines and dioncophyllines in *Ancistrocladus ileboensis* leaves (J. Li et al., 2017d; J. Li et al., 2017c) or xanthohumol and *α*-acid derivatives such as humulones in *Humulus lupulus* flowers (J. Li et al., 2017b; R. Liu et al., 2017). Nevertheless, these compounds might only be slightly different from each other in terms of skeletons as illustrated in the following example: the compounds which structures are shown in **Figure 4** were recently isolated from six different species of *Euphorbia*. They are all diterpenoids that slightly differ in their structures: ent-abietane derivatives (structures Z1 and Z2) (C.-J. Wang et al., 2017), gaditanone (structure AA) (Flores-Giubi et al., 2017), ingenane derivatives (structure AB) (Zhang et al., 2018), premyrsinane and tigliane derivatives (structure AC) (Nothias et al., 2017), dideoxyphorbol ester (structure AD) (Esposito et al., 2017), sooneuphoramine (structure AE) (Gao and Aisa, 2017), jatrophane analog (structure AF) (Rédei et al., 2018), and other abietane derivative (structure AG) (Wei et al., 2017). These compounds all originated from the same biosynthetic pathways where the cyclization reactions of the precursor geranylgeranyl diphosphate and several rearrangements allow many structural variants of diterpenoids to be produced. It is interesting to notice that despite a homology in term of basic scaffold (a phorbol ring system with some rearrangements), all the compounds are extremely different from each other from a chemical point of view.

**Figure 5:**
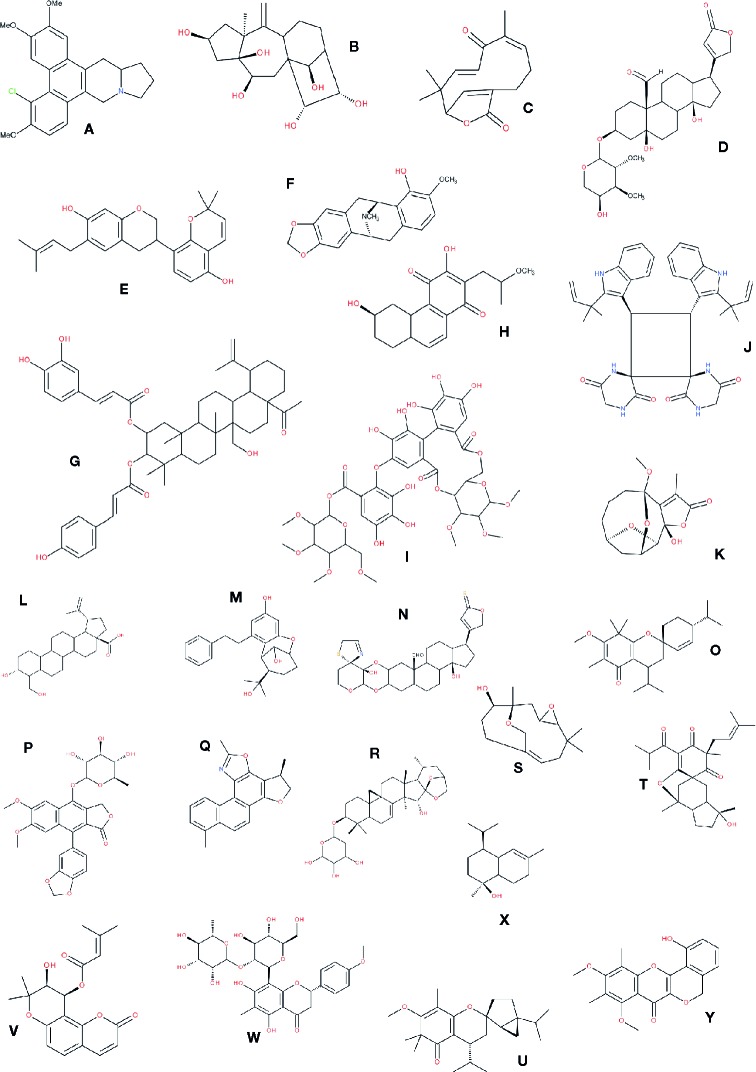
Different types of molecules isolated from randomly chosen plants. Those molecules correspond to examples in **Table 3** where the entries are in bold characters. **(A)** chlorinated phenanthroindolizidine (Al-Khdhairawi et al., 2017); **(B)** 3,4-seco-lupane-type triterpenoid (Cheng et al., 2018); **(C)** asteriscunolide C (Boumaraf et al., 2017); **(D)** (+)-strebloside (Ren et al., 2017); **(E)** Glycybridin D (Li et al., 2017); **(F)** (-)-neocaryachine (Suzuki et al., 2017); **(G)** plectranthroyleanone A (Nzogong et al., 2018); **(H)** uncarilin A (Geng et al., 2017); **(I)** 27-hydroxyalphitolic acid derivative (Novakovic et al., 2017); **(J)** Isorugosin (Shimozu et al., 2017); **(K)** carpescernolide A (Yan et al., 2018); **(L)** 3α,24-dihydroxylup-20(29)-en-28-oic acid (Valencia-Chan et al., 2017); **(M)** rasumatranin A (Wang et al., 2017); **(N)** uscharin (Yoneyama et al., 2017); **(O)** baeckfrutone A (Qin et al., 2018); **(P)** patentiflorin A (Zhang et al., 2017b); **(Q)** salvianan A (Zhang et al., 2017); **(R)** cimiricaside B (Thao et al., 2017); **(S)** aquilanol A (Ma et al., 2017); **(T)** hyperhenone G (Duan et al., 2018); **(U)** frustescone O(Hou et al., 2017); **(V)** dihydropyrano-coumarin derivative (Hong and Kim, 2017); **(W)** matteuorienate A (Huh et al., 2017); **(X)** t-muurolol (Pérez-Colmenares et al., 2018); **(Y)** bougainvinones I (Do et al., 2018). Note: for most of those compounds, the stereospecificity was represented.

This last statement requires underlining that the difference between two chemicals can be dramatic concerning their biological activities while minimal concerning their chemistry. For example, the chemical difference between testosterone and estrone is a saturation of the A cycle of the cholesterol backbone. These minor chemical differences leading to massive differences in pharmacological potential have been the source of a never ending debate among screeners in the pharmaceutical industry on what should populate chemical libraries: should minor variations of basic skeletons be included or not (in the primary screening) knowing that a missing methyl could lead to a nonactivity and *vice-versa*? In our view, it seems important to gather as many compounds as possible inside a chemical series, even if the diversity seems to be futile, because, by setting the minimum results at a poor but significant level, such as 1 to 10 µM (depending on the molecular target) hits could be found, and even minor differences in structures can lead to new leads. In this sense, working with phytochemical diversity as shown in **Figure 5** becomes meaningful.

Furthermore, most of these compounds would be difficult to obtain by conventional organic chemistry methods, not only because of the presence of several intramolecular bridges, but also because of the stereochemistry of the final product [see for example discussion on isoprenoids (Bouvier et al., 2005)]. Secondary metabolites are the results of multienzymatic pathways, and all these enzymes have a strict stereo-specificity. These multiple possibilities in terms of spatial arrangement in compounds contribute to the wide range of pharmacophores lying in natural products.

**Figure 6:**
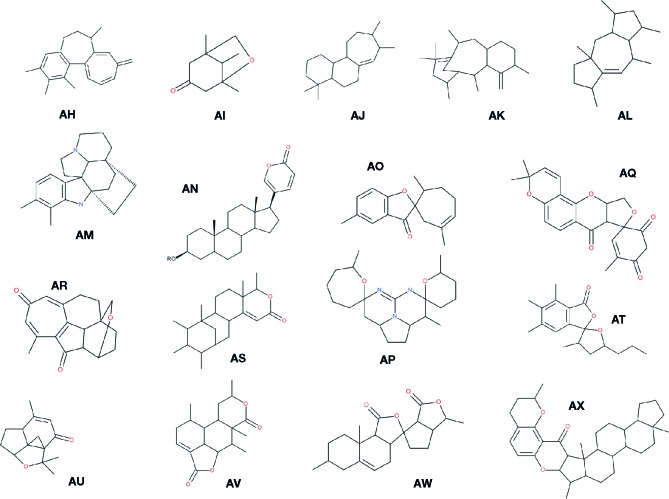
**Examples of skeletons of natural compounds described in the present survey**. The following chemical names were given when the structures were fairly simple. For other more complex skelettons (**AM**, **AP**, **AQ**, **AR**, **AU** and **AV**), common names were given that correspond to the global structure of the core molecule. We simplified the structures in order to give a general idea of the various natural structures met in natural compounds. Many variations exist of those families of compounds. **AH,** 1,2,3,7-tetramethyl-9-methylene-6,7-dihydro-5H-benzo[a]heptalene (Zarev et al., 2017); **AI,** 1,5,8-trimethyl-6-oxabicyclo[3.2.1]octan-3-one (Jiang et al., 2017); **AJ,** 4,4,8,9-tetramethyl-1,2,3,4a,5,6,8,9,10,11,11a,11b-dodecahydrocyclohepta[a]naphthalene (Wang et al., 2017a); **AK,** taxadiene (Dang et al., 2017a); **AL,** 1,3,4,6,8a-pentamethyl-1,2,3,3a,4,6,7,8,9,9a-decahydrocyclopenta [f]azulene (Zheng et al., 2017); **AM,** kopsine (Zeng et al., 2017); **AN,** 5-[(3S,10S,13S,17S)-3-hydroxy-10,13-dimethyl-2,3,4,5,6, 7,8,9,11,12,14,15,16,17-tetradecahydro-1H-cyclopenta[a]phenanthren-17-yl]pyran-2-one (Schwikkard et al., 2017); **AO,** 1′,5,5′-trimethylspiro[benzofuran-2,6′-cycloheptene]-3-one (Luo et al., 2017); **AP**: amorphispirone (Muharini et al., 2017); **AQ,** cephalotane (Zhao et al., 2017); **AR,** xylorumphiin (Waratchareeyakul et al., 2017); **AS,** 5-ethyl-1,8a-dimethyl-6-(1,2,3,4-tetramethylcyclohexyl)-5,6,7,8-tetrahydro-1H-isochromen-3-one (Campos et al., 2017); **AT,** 3′,5,6,7-tetramethyl-5′-propyl-spiro[isobenzo furan-3,2′-tetrahydrofuran]-1-one (Xiao et al., 2018); **AU,** aromaticane (Dong et al., 2017); **AV,** clerodane (Bisio et al., 2017); **AW,** 1,7′,9′a-trimethyl spiro[3a,5,6,6a-tetrahydro-1H-cyclopenta[c]furan-4,3′-4,6,7,8,9,9b-hexahydro-3aH-benzo[g] isobenzofuran]-1′,3-dione (Seeka et al., 2017); **AX,** catechin-bound ceanothane-type triterpenoid (Kang et al., 2017a).

It seems interesting not only to mention the plant chemical diversity but also to show it with some interesting and diverse structures: **Figure 5** already presented about 20 different chemical skeletons of NP that are reported in **Table 4** (compound appearing in bold cases). More diversity is presented in **Figure 6**. To be noticed that compounds issued from microorganisms are often—but not always—peptide-derived structures, often macrocyclic compounds (Newman and Cragg, 2015), such as the families of antibiotics found in *Penicillium* and the like, and thus are not the main purpose here.

However, it is a fact that the structures presented in **Figure 6**, even if they are almost randomly chosen, are different from what provides the current state of the art in medicinal chemistry. This is not really surprising when considering that 83% of core ring scaffolds shown in natural products cannot be found in libraries of synthetic compounds (Harvey et al., 2015). It should be reminded that part of the interest in “discovering” such structures, if they have any biological activities, resides in our capacity to use medicinal chemistry to translate such complex structures in simpler molecules amenable to the industrial production. At the same time, those structures might bear activity towards proteins, the inhibition of which has not been reached yet, with our current access to chemical synthons. This has been reported and discussed according to two points of view: (a) the natural compound-derived fragments (Rodrigues et al., 2016) and (b) the list of compounds issued from natural skeletons: what Newman and Cragg (2016) called NP derivatives in which 268 out of 1,328 new drugs can be found. Among the classical examples, there are vincristine and vinblastine as part of vinca-alkaloids (Zhou and Rahmani, 1992), statins (Sirtori, 2014), glifozins (Burson and Moran, 2015), and ingenol mebutate (Alchin, 2014). It should be reminded here that the current process is moving more from “simple” hits—from whatever origin—to more complex molecules by means of medicinal chemistry decoration of these synthons. The process of lead hopping, as defined by Krueger et al. (2009) and Chakka et al. (2017), should theoretically permit to mimic one complex structure by another, simpler one. All this body of techniques and theories should be put together at work in order to gain new powerful compounds from new, NP-based approaches (Yñigez-Gutierrez and Bachmann, 2019).

In fact, a survey of the data indicates two important features of NP, on the basis of this selection: 1/compounds are very diverse even if they include some common features (like for instance the particular cycloheptanic structure found in some of the main compounds from *Euphorbia*—see **Figure 4**) and 2/the high number of asymmetric carbons render their synthesis by standard organic chemistry difficult if not impossible. For instance, some examples of those numbers are given in **Table 5**. Considering that the number of theoretically possible isomers is 2^n^, n being the number of asymmetric carbons, in some cases, the total number of possible isomers is in the several thousand ranges (see numbers in **Table 5**). Nevertheless, a series of impressive chemical papers reported complete syntheses of such compounds by “standard” synthetic organic chemistry, even if the up-scaling of such *tour de forces* remains to be addressed (Kuttruff et al., 2014).

**Table 5:** Examples of asymmetric carbons in natural products.

Molecules	Number of asymmetric carbons	(Number of possible isomers)	Reference
Bufadienolide	6	(64)	(Schwikkard et al., 2017)
3*α*,24-dihydroxylup-20(29)-en-28-oic acid	7	(128)	(Valencia-Chan et al., 2017)
Xylomexicanin I	7		(Wu et al., 2017)	
Salvinorin A	7		(Yilmaz et al., 2017)	
Sucupiranin A	8	(256)	(Endo et al., 2017)
17-nor-cephalotane-type diterpenoids	9	(512)	(Zhao et al., 2017)
Pharicin C	9		(Hu et al., 2018)	
Scalaradial	9		(Starkus et al., 2017)	
Oridonin	9		(Xu S. et al., 2017)	
Xylorumphiin E	11	(1024)	(Waratchareeyakul et al., 2017)
Cimiricaside B	12	(2048)	(Thao et al., 2017)
Bistinospinoside A	15	(16384)	(Li et al., 2017e)

## Different Strategies to Benefit From This Diversity

As previously mentioned, a deep interest lies in searching and exploring the immense plant chemical diversity for drug discovery purposes, but the strategies to do so need to be reevaluated. Indeed, most of the natural secondary metabolites mentioned herein are not—so far and by far—easily synthesized. It is still through harvesting that we can use plants for discovery and development purposes or for industrial scale production. Compounds with a superior pharmacological activity isolated from a plant part must be isolated from plant extracts where they lay in minor amounts. Certainly, as previously discussed, this is a main bottleneck for many applications, as it is time-consuming, the “superior activity” can vanish in the process for several reasons, or a compound of already known activity can be rediscovered at the end of the process.

In our view these limitations can be overcome by developing alternative strategies. Among these strategies, it seems that the following ones could help solve some of the difficulties linked to NP discovery programs. The first one focuses on gaining systematic access to compounds from plant parts: it deals with the use of scalable cultures of plant cells remaining capable of producing these specific chemicals. It would allow to bypass repetitive massive *in natura* harvests and to have the opportunity to go back to the biological material for further chemical explorations of the remaining compounds, if some of its own limitations would be overcome. The second strategy aims at the appreciation of the known plant chemical diversity, through an “inventory” of NP sources. The third strategy focuses on the industrial scale production through synthetic biology, a branch of biotechnology by which one can construct inside a microorganism the enzymatic pathways leading to a given compound (Zhao et al., 2019). This last body of techniques leads to the possibility to grow thousands of liters of such microorganisms and thus, to obtain large amounts of the desired product(s).

### “Research and Discovery”: *In Vitro* Culture

Regarding plant cell culture, several recent reviews bring a new light to *in vitro* culture, for investigation purposes as well as for its uses as a valuable platform for high-value metabolite production (Wilson and Roberts, 2012; Moscatiello et al., 2013; Ochoa-Villarreal et al., 2015; Eibl et al., 2018). Examples are scattered in the literature with *in vitro* cultures producing compounds of interest either using dedifferentiated cells from callus or undifferentiated cells from meristematic cambial cells. From the calli, systems of plant suspension cell cultures can be generated like for example for acteoside production from *Scrophularia stiata* (Khanpour-Ardestani et al., 2015), rosmarinic acid from *Satureja khuzistanica* (Sahraroo et al., 2014), or carotenoid from *Tagetes erecta* (Benítez-García et al., 2014).

On the other hand, tissue cultures (*i.e.* hairy roots) can also be developed from already differentiated cells. All these types of culture are generally developed for a particular purpose, often very restricted to a given compound in a given plant, such as for camptothecin production by *Ophiorrhiza* species (Asano et al., 2009), *Schisandra chinensis* lignans production (Szopa et al., 2017; Szopa et al., 2018) or boeravinone Y by *Abronia nana* (Lee et al., 2018). Several examples of cultures at a commercial scale have also been described validating its feasibility and scalability from lab-scale to large-scale (paclitaxel form *Taxus* spp. cultures, rosmarinic acid from *Coleus blumei* cultures, scopolamine from *Duboisia* spp. cultures (Wilson and Roberts, 2012) to name only but a few).

Nevertheless, if the literature provides us with some very well-described examples of such tasks, it remains to be seen if these techniques are universal. In other words, what has been described to be possible to obtain a “large scale” plant cell culture is not necessarily applicable to the next cell culture of a different plant, and *a fortiori*, of a different organ of the same plant species. What has been considered as a promising approach remains in some cases a challenge, as no experimental process has been developed—or published—with a general usage purpose. Thus, the perfectly described process to obtain stem-derived callus or leaf-derived callus producing anticancer phenolic compounds from *Fagonia indica* (Khan et al., 2016) is only partially similar to the process to obtain callus from fruit pulp of varieties of apple producing high triterpenic acids (Verardo et al., 2017) or the process to obtain callus originating from seeds of *Abronia nana* (Kim et al., 2014), a desert plant found in North America to produce massive amount of boeravinone Y (Lee et al., 2018) or even callus from *Scrophularia striata* for the production of acteoside (Khanpour-Ardestani et al., 2015). As seen above, many examples can be found in the literature, but the remaining question is how much the methodology varied from one example to the next in order to obtain such callus and then to obtain such cell suspensions producing the desired compound. Some general procedures for the establishment of dedifferentiated plant cell suspension cultures exist (Mustafa et al., 2011; Eibl et al., 2018) but with many specific adaptations in the function of species, organs of origins, and culture conditions. Probably because the authors concentrated mainly on the productivity and yield of the targeted compound and not on a larger picture applicable to a more general view of accessing and testing the chemical diversity of plants. If one considers the natural diversity lying in the plant species from our environment as a source of “new” chemicals, it would be wonderful to rely on a methodology universal enough to collect the biological material just once (or a very few times) and then, to rationalize the culture of the cells originating from the specific plant organ (leaves, stems, roots, *etc*.). The interest on these cell cultures is that the cells would be able to biosynthesize a large variety of compounds in quantities large enough to isolate and identify compounds with pharmacological activity and completely characterize them. At that stage, the culture size can be customized by expanding from several liters to several tens of liters of cell culture (hundreds if the initial results are promising and more biomass is needed to go on with testing). Finally, even modifications of culture conditions could work at enhancing chemical diversity (Jozwiak et al., 2013) or at least variations within the proportions of different secondary metabolites (Jozwiak et al., 2013; Akbari et al., 2018; Saad et al., 2018).

The originality of our approach thus resides in using plant tissue and cell culture not for the production scale for which some limitations exist but to attempt facilitating the access to plant chemical diversity. With these perspectives, a repository associated with plant cell culture would be a valuable tool. And the versatility in terms of scaling the *in vitro* cultures would also allow bridging the gap between drug discovery and the first stages of development.

### At the Development Stage: Systematic Inventories of Natural Products and Their Sources

Classically, the approaches used for drug discovery—detailed above for some of them—are quite specific: trying to find a compound in a plant organ that has some specific activity against a particular enzyme, receptor, or pathway. On the contrary, a systematic “inventory” of the NP existing in living organisms, in plant parts for example, would be of great interest both for the drug discovery aspects as well as the development aspects, for the discovery aspects because it would allow a better use of the known natural chemical diversity and for the drug development aspects, because it would allow to change the sourcing of the NP keeping in mind how important the supply of the compound of natural origin is for a company.

As shown for a few examples in **Tables 2** **–** **4** which consist in a sampling of recently reported works on natural compounds, this “Systematism” is already used by a few groups who catalogued the chemical compounds in a given organ of a given plant (Batista et al., 2017; Sendker et al., 2017; Ma et al., 2018; Sharma et al., 2018), in a fungus (Chang et al., 2017), or in a microorganism (McMullin et al., 2017; Verastegui-Omana et al., 2017). In such cases, an idea of the possible diversity of those sources is given. A compendium of some plant compositions had been done by I.A. Ross (Ross, 1999; Ross, 2001). Such inventory organized in a global database would greatly facilitate the access to the diversity of secondary metabolites of plants, for example. It would somewhat ease any strategy based on chemotaxonomy by describing better the filiations/relationships between the biosynthetical pathways in a different genus and by facilitating the access to some types of chemical skeletons in renewable naturally producing sources such as leaves or fruits. Furthermore, **Table 4** indicates, in our view, the way the literature can be compiled from all the available sources to build a database based only on published articles describing one or several compounds from plant parts. In this line, the remarkable paper of Solyomváry et al. (2017) reviews the available literature on the compounds from the dibenzylbutyrolactone lignan family and describes some 91 compounds of this chemical family from their origin in terms of plant species and plant parts.

For many reasons, such a systematic inventory of plants chemical components related to the tissues and the species from which they have been extracted, would be of great use but could be difficult to complete, even with modern and fast analytical tools. Indeed, it is the completion of such a compendium that is the real challenge, and the experience of the few already existing NP databases exemplifies that challenge. For example the NAPRALERT experience (https://napralert.org) gathering data from more than 200,000 scientific papers is very informative: comprehensive coverage is claimed from 1975 to 2004 while only 20% of the global data is covered from 2005 due to budgetary constraints. Organisms, compounds, activities, or authors can be searched. In fact, the last decade has seen the development of several databases providing systematic collection of information that focuses on natural compounds themselves, offering the possibility of searching structure, source, and mechanisms of action of the searched compounds. For example, DEREP-NP is a database that compiles structural data (Zani and Carroll, 2017). An interesting review from Xie et al. (2015) allows the comparison of fourteen of these databases focusing on NP, balancing their advantages and disadvantages. Among them, the updated version of a 2006 database SuperNatural II is a public resource (http://bioinformatics.charite.de/supernatural) with more than 325,500 natural compounds, offering 3D structure and conformers (Banerjee et al., 2015) which seems to outperform many others (Harvey et al., 2015; Xie et al., 2015). Another source of natural compounds is also the Greenpharma collection (www.greenpharma.com/products/compound-librairies/#GPNDB) (Do et al., 2015; Gally et al., 2017).

### Industrial Scale Production: Synthetic Biology and Organic Syntheses

The use of plant cell and organ culture for the production at the industrial scale of compounds with superior added value has been reported in various reviews (Wilson and Roberts, 2012; Imseng et al., 2014; Eibl et al., 2018). These reviews cited a large range of applications from the pharmaceutical area (suspension cells of Pacific yew in 75m^3^ stirred bioreactors delivering 500kg/year of paclitaxel) to the cosmetic and food industries like cell cultures of *Malus domestica* grown in 50 to 100 L production bioreactors. But still, it is acknowledged that some limitations exist: mainly the fact that time-consuming processes are involved, with possibly low titers, and the possibility of somaclonal variations appearing in the selected top producing cell lines. Several solutions to try to avoid these kinds of limitation can be assessed (Trosset and Carbonell, 2015), but in our opinion, general strategies should consider other alternatives for the large industrial production scale depending on the kind of applications. For some specific NP of pharmacological interest like podophyllotoxin, artemisinin or plumbagin for example, a whole set of biotechnological approaches has been developed and described for the production at larger scale of these valuable compounds (Lautié et al., 2010; Kayani et al., 2018; Roy and Bharadvaja, 2018). But once more, these strategies are quite specific, driven only in one identified compound and its specific biosynthetical pathway.

#### Synthetic Biology

As previously mentioned, one of the major requirements to this approach is to understand the pathways through which a particular compound is biosynthesized, thanks to the activity of a series of enzymes involved in a particular plant part. Within this last approach, the focus in plants is more on their capacity to synthetize unique scaffolds than on the end-products themselves. Indeed, the use of the recent integrative approaches based on “Omic” analyses (metabolomics, proteomics, transcriptomic, and genomics) can be of great value. Indeed, knowing the precursors and intermediates through the biochemical status of a tissue, identifying the key enzymes and the limiting steps of the pathways, monitoring indirectly the function of the genes involved in these pathways and their regulation will contribute to decipher the biosynthetic routes *in planta* (Cheallaigh et al., 2018; Scossa et al., 2018). The relative ease at which one can now obtain large-scale data has facilitated the analyses at the level of the whole metabolic network (Paddon and Keasling, 2014; Ikram and Simonsen, 2017). For example, large amounts of transcriptomic data are now easier to access as stated by Owen et al. (2017), making possible the identification of multistep pathways by coexpression analyses or untargeted metabolomics. Furthermore, the discovery that genes linked to biosynthetic pathways are organized in clusters has opened new opportunities by adapting methodologies developed initially for microorganisms to plants like systematic cluster mining algorithms (Owen et al., 2017). Scossa et al. (2018) reviewed recently the progresses made in the understanding of plant biosynthetic pathways with the integration of metabolomics and next-generation sequencing based on various families of compounds: for example, benzoisoquinoline and monoterpenoid indole alkaloids, cannabinoids, ginsenosides, or withanolides. They also emphasized the new insight that this area can bring in the field of synthesis of NP. They mention for example, the intriguing case of caffeine biosynthesis that evolved independently in several orders of eudicots: at least three metabolic pathways evolved separately coopting genes from different gene families illustrating how biosynthetic pathways can evolve with land plant diversification (Scossa et al., 2018).

After having identified the genes involved, the reconstitution of the biosynthetic pathways of interest can be realized thanks to novel DNA construction technologies. It can be realized in a foreign host which enables the increase of product yields. The choice of this host organism is key as the goal is to develop an efficient platform for heterologous gene expression. Microbial hosts are generally considered more amenable than plants to fermentation process (Atanasov et al., 2015). Among them, classical work horses like *E. coli* or *S. cerevisiae*, or newcomers like *Bacillus subtilis* (Abdallah et al., 2019) and *Pseudomonas putidaare* (Nikel et al., 2014; Loeschcke and Thies, 2015; Choi and Lee, 2020) can be cited. Another interesting example is the recent high-cell-density fermentation strategies developed for heterologous production in *Pichia pastoris* (W.-C. Liu et al., 2019). Then, basically, the strategy will consist in cloning the genes of the enzymes of the pathway that have been identified; constructing large plasmid (or family of plasmids) encoding for those enzymes; transfecting with the plasmid a microorganism that will be grown afterwards; and purifying the product (Xiao et al., 2018). Several recent reviews detail how the technical advances in synthetic biology and multiplexed genome engineering allow for optimizing the design and synthesis of the pathways involved in NP production (Awan et al., 2016; Breitling and Takano, 2016; Carbonell et al., 2016; Smanski et al., 2016; Moses et al., 2017). Many such examples can be found such as for curcumin synthesis reconstitution in *E. coli* (Kang et al., 2018), polyunsaturated fatty acids production in the fungus *Ashbya gossypii* (Ledesma-Amaro et al., 2018), *α*-amyrin, lupulones ou ginsenosides synthesis in *S. cerevisiae* (Dai et al., 2014; Yu et al., 2018; Guo et al., 2019), or the diversification of the carotenoid biosynthetic pathways (Umeno et al., 2005). But probably the best example of economically feasible process is reported for the production of artemisin at an industrial scale (Paddon and Keasling, 2014; Ikram and Simonsen, 2017).

Alternatively, the developments in plant transformation and transfection technology offering rapid and scalable biosynthesis allow for considering more and more the use of plant-based expression platforms like *Nicotiana* or *Arabidopsis* spp.(Fuentes et al., 2016; Lu et al., 2017; Reed et al., 2017; Appelhagen et al., 2018). Indeed they are considered genetically more flexible than the native plant sources and offer in some cases several advantages even over microbial hosts that can lack the endogenous biosynthetic precursors of these NP or intracellular compartments as endoplasmic reticulum related with the implementation of enzymes like cytochrome P450s (Appelhagen et al., 2018).

These advances in plant synthetic biology will increase the access to NP through new synthetic routes (Reed et al., 2017) but will also allow the synthesis of new-to-nature molecules and so, expand the natural plant chemical diversity.

#### Organic Syntheses

A way to analyze the total syntheses that have been recently produced in the literature is to use simple criteria in order to evaluate the feasibility of such approach in case of similar compounds finding their way to the clinic. We evaluated a set of recent publications dealing with “total synthesis” of NP according to the simple criteria: number of cycles in the compounds, number of carbons and heteroatoms—including sulfur—in those cycles and number of asymmetric carbons in these structures (**Table 6**). In this nonexhaustive set of publications, it was decided not to consider peptides and peptide-derived macrocycles (about a dozen structures). The next observation was that there were a surprising high number of bacteria-derived compounds, a feature that we did not notice in our previous surveys (**Tables 2** **–** **4**). Another parameter that allows for judging the feasibility and scalability of the processes is the yield and the number of steps. In that sense, most of those works are exquisitely delicate enterprises. The success of those publications in terms of *tour de force* is obvious, but they also allow for emphasizing the necessity to obtain such general synthetic routes, as most of those were used then to provide analogs to the desired NP in each publication.

**Table 6:** Some examples of total synthesis of natural products.

Compound (reference)	Origin*	Number of cycles	Number of carbons **	Number of heteroatoms**	Number of asymetric centers	number of steps***	yield (%)***
cerorubenic acid III (Liu et al., 2019)	*Ceroplastes rubens (insect)*	4	15	0	7	~20	
(−)-ambiguine P (Johnson et al., 2019)	*Fischerella ambigua (cyanobacteria)*	5	18	1	4	20	
actinorhodin (Ninomiya et al., 2019)	*Streptomyces coelicolor (actinobacteria)*	2 × 3	2 × 13	2 × 1	2 X1	16	1,6
(+)-nivetetracyclate A (Blitz et al., 2019)	*Streptomyces niveus (actinobacteria)*	4	18	0	3	17	< 2
nogalamycin (Peng and VanNieuwenhze, 2019)	*Streptomyces nogalator (actinobacteria)*	6	23	2	5	> 20	
albomycins δ1 (Lin et al., 2018)	*Streptomyces griseus (actinobacteria)*	2	8	3	10	13	
(−)-omuralide (Rulliere et al., 2018)	*Streptomyces lactacystenaeus (actinobacteria)*	2	5	3	4	19	2,6
(+)-guadinomic acid (Reid et al., 2018)	*Streptomyces* sp. *(actinobacteria)*	1	3	2	2	7	0,6
(−)-(3R)inthomycin C (Balcells et al., 2018)	*Streptomyces* sp. *(actinobacteria)*	1	3	2	2	10	15
(±)-vibralactone (Nistanaki et al., 2019)	*Boreostereum vibrans (fungus)*	2	6	1	2	6	4,3
*epi*-trichosetin (Kauhl et al., 2018)	*Fusarium oxysporum (fungus)*	3	15	1	6	8	4,1
tryptoquivaline (Wei and Dixon, 2018)	*Aspergillus fumigatus (fungus)*	5	19	3	2	~20	11
(−)-chaetominine (Geng and Huang, 2019)	*Chaetomium* sp. *(fungus)*	6	21	4	4	4	33,4
repraesentin F (Ferrer and Echavarren, 2018)	*Lactarius repraesentaneus (fungus)*	3	10	0	6	16	2
epicolactone (Kravina and Carreira, 2018)	*Epicoccum nigrum (fungus)*	5	15	2		17	
(−)-6,7-dideoxysqualestatin H5 (Almohseni et al., 2018)	*Lepzodontium elarius (fungus)*	3	12	2	1	11	5,5
delitschiapyrone A (Kurasawa et al., 2018)	*Delitschia* sp. *(fungus)*	5	18	2	5	8	33
phyllostictine A (Riemer et al., 2018)	*Phyllosticta cirsii (fungus)*	2	7	2	3	13	4
spiromamakone A (Tsukamoto et al., 2018)	*Preussia* sp. *(fungus)*	4	19	0	1	16	
penicophenone A (Pantin et al., 2018)	*Penicilium dipodomycola (fungus)*	3	13	2	3	14	6
aurofusarin (Qi et al., 2018)	*Fusarium graminearum (fungus)*	2 x 3	26	2 x 1	0	10	10
(±)-antroquinonol (Wang et al., 2019)	*Antrodia camphorata (mushroom)*	1	6	0	3	10	29
Suillusin (Zhang et al., 2018)	*Suillus granulatus (mushroom)*	4	17	1	2	8	11
conosilane A (Yuan et al., 2018)	*Conocybe siliginea (mushroom)*	4	13	2	4	10	
Isopanepoxydone (Man et al., 2018)	*Lentinus strigellus (mushroom)*	2	6	1	3	9	21
cochlearoid B (Zhang et al., 2018)	*Ganoderma cochlear (mushroom)*	5	18	2	3	7	12
boletopsin 11 (Zhang and Barrow, 2018)	*Boletopsis* sp. *(mushroom)*	4	18	1	0	9	6
(+)-dimericbiscognienyne A (Kim et al., 2018)	*Biscogniauxia* sp. *(mushroom)*	7	18	3	12	7	
(−)-mitrephorone A (Richter et al., 2018)	*Mitrephora glabra (tree)*	5	17	1	5	7	26
(+)-chamuvarinin (Samala et al., 2018)	*Uvaria chamae (plant)*	3 × 1	12	3	7	20	3
(±)-aspidofractinine (Saya et al., 2018)	*Aspidosperma cylindrocarpon (tree)*	6	17	2	4	8	< 5
(+)-leucomidine A (Yao et al., 2018)	*Leuconotis griffithii (plant)*	5	17	3	2	8	31
larreatricin 1 (Martin et al., 2018)	*Larrea tridentata (plant)*	3	16	1	4	6	40
(±)−exotine B (Cheng et al., 2018)	*Murraya exotica (plant)*	5	22	2	2	6	
lanceolactone A (Acharyya and Nanda, 2018)	*Illicium lanceolatum (plant)*	2	7	2	2	4	44
bussealin E (Twigg et al., 2018)	*Bussea sakalava (plant)*	4	15	1	0	11	14
polyflavanostilbene B (Wang X. et al., 2018)	*Polygonum cuspidatum(plant)*	9	40	2	7		
“Unnamed alkaloid” (Davison et al., 2018)	*Isatis indigotica (plant)*	4	14	5	2	6	
2-epi-narciclasine (Borra et al., 2018)	*Narcissus* sp. *(plant)*	4	13	3	4	9	4,5
(+)-psiguadial B (Chapman et al., 2018)	*Psidium guajava (plant)*	6	23	1	7	15	1,3
Parvineostemonine (Gerlinger et al., 2018)	*Stemona parviflora (plant)*	4	14	2	4	5	17
Arboridinine (Gan et al., 2018)	*Kopsia arborea (plant)*	5	16	2	3	16	
adunctin B (Dethe and Dherange, 2018)	*Piper adunctum (plant)*	4	18	1	3	6	23
(±)-deguelin (Xu et al., 2018)	*Tephrosia vogelii (plant)*	5	19	3	2	4	62
englerin A (Hatakeyama, 2018)	*Phyllanthus engleri (plant)*	5	19	2	6	23	13
houttuynoid A (Jian et al., 2018)	*Houttuynia cordata (plant)*	5	22	3	4	7	24
(−)-mucosin (Nolsoe et al., 2018)	*Reniera mucosa (sponge)*	2	8	0	2	15	
(*rac*)-renieramycin T (Kimura and Saito, 2018)	*Reniera* sp. *(sponge)*	6	20	4	5	19	1,8
(+)-frondosin B (Zong et al., 2018)	*Dysidea frondosa (sponge)*	4	16	1	1	15	12
shishijimicin A (Nicolaou et al., 2018b)	*Theonella* sp. *(sponge)*	7	36	4	8	> 20	16
ulbactin F (Shapiro et al., 2018)	*Brevibacillus* sp. *(sponge)*	5	16	6	5	7	12
spongosoritin A (Alcock et al., 2018)	*Spongosorites* sp.*(sponge)*	1	4	1	2	11	1,8
Namenamicin (Nicolaou et al., 2018a)	*Polysyncraton lithostrotum (ascidian)*	5	29	3	11	> 20	14

*The origin of the compounds is color-coded: light red: insect; black: bacteria; light blue: fungus; violet: mushroom; green: plants and trees; dark red, sponge; dark blue: ascidian. ** The numbers of carbon atoms and of heteroatoms were those included in the cycles. ***Yellow cells: approx. calculations by the present authors. Gray cells: This information is too deeply embedded in the paper.

Some of those compounds are devoid of asymmetric carbons, rendering the synthesis ‘easier’, but still a challenge requiring several steps, with an overall poor yield. At the other end of the spectrum are compounds with a considerable number of asymmetric carbons, such as (+)-dimericbiscognienyne A with 12 asymmetric carbons (Kim et al., 2018), or namenamicin with 11 (Nicolaou et al., 2018a) and/or with a high number of cycles, even if they were not always fused with each other. Indeed, a series of three furan-based cycles (Samala et al., 2018) separated by alkyl carbon chains would not be a considerable difficulty to synthesize, depending on the decorations of those cycles that introduce notions of asymmetries and thus difficulties to perform.

Those data, when compared to the ones gathered in **Table 5**, show that in these particular cases, the access by chemistry of all the possible optical isomers would be simply impossible. These observations cast some shadows on the possibility of using those synthetic routes at the industrial scale. On the other hand, the mastering of some steps, particularly the stereo-controlled ones, are key in the cases where alternative hemi-syntheses solutions are adopted from a most abundant intermediary (natural) compound. Finally, another point is certainly the growing numbers of synthetic routes that are explored, assessed, and validated to access some “common” features from those natural compounds. A review of this literature can be found in Li L. et al. (2018). Even if partial by essence, it shows the considerable number of routes that has been set up and that permits access to some of the main fused cycles found in compounds coming from different natural sources. Another review summarized the way spiroacetal can be accessed, another common feature of many natural compounds (Zhang et al., 2018). This last point strongly emphasizes the common nature throughout the living world of the basic enzymatic systems aiming at producing secondary metabolites from the same fundamental bricks such as mevalonate or other isoprenoids.

Nevertheless, it is also clear from that survey that chemistry is not, at the present time, the solution to the problem of scalability of NP productions to an industrial level, even if these compounds were extremely active on a given disease and even if a large panel of examples in which complete syntheses of NP are presented (Kuttruff et al., 2014).

As pointed out earlier in the present assay, at the research level and even at the level of exemplifications of chemical analogs of a given active, these approaches are necessary and important. Indeed, deciphering the various routes to some of those compounds might help design and simplify the overall structures, as it is the case in standard, organic chemistry-based, medicinal chemistry.

## Conclusions

For decades, the interplay between the search for “new” drugs and NP has been strong, to a point where some fear that the destruction of native forests, leading to a reduction of plant diversity would jeopardize our finding of new cures for old and new diseases. The present essay aimed to offer a global overview of the extent of the known chemical diversity, its access, and its use. Several approaches to chemical diversity were also discussed maximizing, in our view, the possibilities of finding useful compounds for human unmet medical needs.

As illustrated, plant natural chemical diversity is indeed immense. And the knowledge we gathered on plants is only the tip of the iceberg as exemplified in IA Ross’s books (Ross, 1999; Ross, 2001), in which he gathered all the chemical components found in some 40 plants and their many different components. And this knowledge is certainly scattered all over the world. A unifying work should be done, under a simple format, that could be like the one presented in **Table 3** and completed by following the example of Solyomváry et al. (2017). This kind of tool would incredibly ease the access to plant natural chemical diversity and should ideally be comprehensive, organized and include data from worldwide plant species, from past to recent studies. Such a globalized database could furthermore be integrated to other ones like genomic, phylogenic, species occurrence, biosynthetic pathway, biological activity, or chemical classification (Allen et al., 2019) allowing researchers to mine the resources and correlate the information, hence empowering all kind of research studies. This trend has been emphasized by several authors in their recent reviews (Atanasov et al., 2015; Harvey et al., 2015) stating that drug discovery from plants requires multidisciplinary approaches. Experiences from the past tell us how important it is, for drug discovery purposes, to access this wide diversity lying in the Plant kingdom, especially because it may be shrinking due to the rapid alterations of the biosphere. In order to fully access the whole chemical diversity without jeopardizing plant biodiversity, alternative ways to collect and store plant tissues can be explored, as for example the use of *in vitro* culture techniques allowing a renewable and sustainable access to plant chemical diversity. As the final purpose is giving access to workable quantities of therapeutic compound(s), we suggested that the advances in synthetic biology coupled with genomics and bioinformatics can pave the way to possible future strategies of productions of the compounds originating from this diversity. But the chemical diversity in the scaffolds of plant natural compounds is so wide that there is still some space from different strategies for large-scale production: from organic total synthesis for the simpler scaffolds like ephedrine or metformin that are able to be synthetized in few steps, that is to say, at a reasonable cost or, at the other end of the spectrum, heterologous (plant)? production for compounds with more complex scaffolds like taxanes and multistep biosynthesis, and in between even hybrids (multihosts)? semisynthetic strategies can be imagined and developed.

## Author Contributions

EL and JB wrote the review with the help of PD (modelization) and OR (chemistry).

## Conflict of Interest

The authors declare that the research was conducted in the absence of any commercial or financial relationships that could be construed as a potential conflict of interest.
