# Supplementary material for: Unraveling Plant Natural Chemical Diversity for Drug Discovery Purposes
Source: Front Pharmacol. 2020 Apr 7;11:397. doi: 10.3389/fphar.2020.00397 (PMC7154113; doi:10.3389/fphar.2020.00397)
Supplement: Supplementary file 1 [file Table_1.pdf]

Unraveling plant natural chemical diversity for drug discovery purposes

Emmanuelle Lautié[£], Olivier Russo[‡], Pierre Ducrot[$], Jean A. Boutin[‡,*]

[£] Centro de Valorização de Compostos Bioativos da Amazônia (CVACBA) - Instituto de Ciências Biológicas, Universidade Federal do Pará (UFPA), Belém - Pará - Brasil

[‡] Institut de Recherches Internationales SERVIER, Suresnes, France

[$] Institut de Recherches SERVIER, Croissy-sur-Seine, France

*: Author to whom correspondence should be addressed: Jean A. Boutin, Institut de Recherches Internationales Servier, 50 rue Carnot, 92284 Suresnes Cedex, France;

email: ja.boutin.pro@gmail.com

Tel +33 1 55724400

Supplemental table S1: Correspondance of latin names of plants ith the accepted plant species names

	Plant species names as reported in the referenced publication (alphabetical order)	Location in our review	Accepted names following the agreed taxonomy as described in (a): mpns.science.kew.org and (b): www.theplantlist.org		Frequency of appearance in plant medicinal literature
1	*Abronia nana*	Section 3.1	*Abronia nana* S.Watson	(b)	5 (PubMed)
2	*Acacia ligulata*	Table 4	*Acacia ligulata* A.Cunn. ex Benth.	(a)	9
3	*Aconitum apetalum*	Table 4	*Aconitum apetalum* (Huth) B.Fedtsch	(b)	3 (PubMed)
4	*Acorus tatarinowii*	Table 4	*Acorus calamus var. angustatus* Besser	(a)	28
5	*Aframomum melegueta*	Table 1	*Aframomum melegueta* K.Schum.	(a)	35
6	*Allanblackia floribunda*	Table 4	*Allanblackia floribunda* Oliv.	(a)	16
7	*Alnus viridis*	Table 4	*Alnus alnobetula subsp. alnobetula*	(a)	10
8	*Alpinia officinarum*	Table 1	*Alpinia officinarum* Hance	(a)	90
9	*Althaea officinalis*	Table 4	*Althaea officinalis L.*	(a)	202
10	*Amorpha fruticosa*	Table 4	*Amorpha fruticosa L.*	(a)	2
11	*Anabasis articulata*	Table 4	*Anabasis articulata* (Forssk.) Moq.	(a)	14
12	*Ancistrocladus ileboensis*	Table 4	*Ancistrocladus ileboensis* Heubl, Mudogo & G.Bringman	(b)	2 (PubMed)
13	*Anigozanthos rufus*	Table 4	*Anigozanthos rufus* Labill.	(b)	2 (PubMed)
14	*Anthemis nobilis*	Table 4	*Chamaemelum nobile (L.)* All.	(a)	24
15	*Aphanamixis polystachya*	Table 4	*Aphanamixis polystachya* (Wall.) R.Parker	(a)	9
16	*Aquilaria malaccensis*	Table 4	*Aquilaria malaccensis Lam.*	(a)	96
17	*Aquilaria sinensis*	Table 4	*Aquilaria sinensis* (Lour.) Spreng.	(a)	43
18	*Aristolochia orbicularis*	Table 4	*Aristolochia orbicularis* Duch.	(b)	3 (PubMed)
19	*Artocarpus rigida*	Table 4	*Artocarpus rigida* Blume (unresolved)	(b)	5 (PubMed)
20	*Atalantia monophylla*	Table 4	*Atalantia monophylla DC.*	(a)	8
21	*Azadirachta indica*	Table 4	*Azadirachta indica* A.Juss.	(a)	872

22	*Baeckea frutescens*	Table 4	*Baeckea frutescens L.*	(a)	4
23	*Balthasaria mannii*	Table 4	*Balthasaria mannii* (Oliv.) Verdc	(b)	no data
24	*Belamcanda chinensis*	Table 4	*Iris domestica (L.)* Goldblatt & Mabb.	(a)	53
25	*Berchemia berchemiifolia*	Table 4	*Berchemiella berchemiifolia* (Makino) Nakai	(b)	2 (PubMed)
26	*Betula pendula*	Table 4	*Betula pendula* Roth	(a)	86
27	*Betula pubescens*	Table 4	*Betula pubescens* Ehrh.	(a)	35
28	*Boesenbergia pandurata*	Table 4	*Boesenbergia rotunda* (L.) Mansf.	(a)	8
29	*Bougainvillea spectabilis*	Table 4	*Bougainvillea spectabilis* Willd.	(a)	21
30	*Bowdichia virgilioides*	Table 4	*Bowdichia virgilioides* Kunth	(a)	14
31	*Buddleja asiatica*	Table 4	*Buddleja asiatica* Lour.	(a)	27
32	*Bupleurum fruticosum*	Table 4	*Bupleurum fruticosum L.*	(a)	1
33	*Calotropis gigantea*	Table 4	*Calotropis gigantea* (L.) W.T.Aiton	(a)	56
34	*Camellia crapnelliana*	Table 4	*Camellia crapnelliana* Tutcher	(b)	3 (PubMed)
35	*Carpesium cernuum*	Table 4	*Carpesium cernuum L.*	(a)	2
36	*Caryopteris nepetaefolia*	Table 4	*Caryopteris nepetifolia* (Benth.) Maxim.	(b)	1 (PubMed)
37	*Catalpa ovata*	Table 4	*Catalpa ovata* G.Don	(a)	15
38	*Celastrus subspicata*	Table 4	*Celastrus subspicatus* Hook.	(b)	1 (PubMed)
39	*Cephalotaxus fortunei*	Table 4	*Cephalotaxus fortunei* Hook.	(b)	26 (PubMed)
40	*Cephalotaxus sinensis*	Table 4	*Cephalotaxus sinensis* (Rehder & E.H.Wilson) H.L.Li	(b)	9 (PubMed)
41	*Ceratodon purpureus*	Table 4	*Ceratodon purpureus* (Hedw.) Brid.	(b)	90 (PubMed)
42	*Chaenomeles sinensis*	Table 4	*Chaenomeles sinensis* (Dum.Cours.) Koehne	(a)	23
43	*Chrysanthemum indicum*	Table 4	*Chrysanthemum indicum L.*	(a)	44
44	*Chrysanthemum morifolium*	Table 4	*Chrysanthemum* × *morifolium* (Ramat.) Hemsl.	(a)	71
45	*Cimicifuga dahurica*	Table 4	*Actaea dahurica* (Turcz. ex Fisch. & C.A.Mey.) Franch.	(a)	31
46	*Cinnamomum cassia*	Table 4	*Cinnamomum cassia* (L.) J.Presl	(a)	280
47	*Citrus maxima*	Table 1	*Citrus maxima* (Burm.) Merr.	(a)	52
48	*Citrus paradisii*	Table 1	*Citrus* × *aurantium* L.	(a)	6
49	*Citrus sinensis*	Table 1	*Citrus sinensis* (L.) Osbeck	(b)	2538 (PubMed)
50	*Clausena anisumolens*	Table 4	*Clausena anisata* (Willd.) Hook.f. ex Benth.	(b)	7 (PubMed)
51	*Cleistochlamys kirkii*	Table 4	*Cleistochlamys kirkii* (Benth.) Oliv.	(b)	5 (PubMed)

52	*Codonopsis pilosula*	Table 4	*Codonopsis pilosula* (Franch.) Nannf.	(a)	67
53	*Coleonema album*	Table 4	*Coleonema album* (Thunb.) Bartl. & H.L.Wendl.	(a)	2
54	*Coleus blumei*	Section 3.1	*Plectranthus scutellarioides* (L.) R.Br.	(a)	1
55	*Cornus officinalis*	Table 4	*Cornus officinalis* Siebold & Zucc.	(a)	60
56	*Curcuma aromatica*	Table 4	*Curcuma aromatica* Salisb.	(a)	191
57	*Curcuma longa*	Table 4	*Curcuma longa L.*	(a)	437
58	*Cyclopia intermedia*	Table 4	*Cyclopia intermedia* E.Mey.	(a)	13
59	*Cynomorium songaricum*	Table 4	*Cynomorium coccineum subsp. songaricum* (Rupr.) J.Léonard	(a)	38
60	*Dasymaschalon echinatum*	Table 4	*Dasymaschalon echinatum* Jing Wang & R.M.K.Saunders	(b)	no data
61	*Dennettia tripelata*	Table 1	*Dennettia tripetala* Baker f.	(b)	16 (PubMed)
62	*Eremophila longifolia*	Table 4	*Eremophila longifolia* (R.Br.) F.Muell.	(a)	3
63	*Erythrina schliebenii*	Table 4	*Erythrina schliebenii* Harms	(b)	4 (PubMed)
64	*Eugenia jambolana*	Table 1	*Syzygium cumini* (L.) Skeels	(a)	3
65	*Euphorbia ebracteolata*	Table 4	*Euphorbia ebracteolata* Hayata	(a)	8
66	*Euphorbia fischeriana*	Table 4	*Euphorbia fischeriana* Steud.	(a)	13
67	*Euphorbia gaditana*	Table 4	*Euphorbia gaditana* Coss	(b)	1 (PubMed)
68	*Euphorbia kansui*	Table 4	*Euphorbia kansui* S.L.Liou ex S.B.Ho	(a)	27
69	*Euphorbia pithyusa*	Table 4	*Euphorbia pithyusa L.*	(b)	9 (PubMed)
70	*Euphorbia semiperfoliata*	Table 4	*Euphorbia semiperfoliata* Viv.	(b)	7 (PubMed)
71	*Euphorbia soongarica*	Table 4	*Euphorbia soongarica* Boiss.	(a)	1
72	*Euphorbia taurinensis*	Table 4	*Euphorbia taurinensis* All.	(b)	2 (PubMed)
73	*Excoecaria agallocha*	Table 4	*Excoecaria agallocha L.*	(a)	11
74	*Fagonia indica*	Section 3.1	*Fagonia indica* Burm.f.	(a)	13
75	*Ficus fistulosa*	Table 4	*Ficus fistulosa* Reinw. ex Blume	(a)	3
76	*Fissistigma latifolium*	Table 4	*Fissistigma latifolium* (Dunal) Merr.	(a)	1
77	*Flindersia pimenteliana*	Table 4	*Flindersia pimenteliana* F.Muell.	(b)	2 (PubMed)
78	*Forsythia suspensa*	Table 4	*Forsythia suspensa* (Thunb.) Vahl	(a)	63
79	*Fraxinus angustifolia*	Table 1	*Fraxinus angustifolia* Vahl	(a)	30
80	*Garcinia propinqua*	Table 4	*Garcinia propinqua* Craib	(b)	5 (PubMed)
81	*Gardenia ternifolia*	Table 4	*Gardenia ternifolia* Schumach. & Thonn.	(a)	21

82 *Gloriosa superba*	Table 4	*Gloriosa superba L.*	(a)	208
83 *Glycyrrhiza glabra*	Table 1	*Glycyrrhiza glabra L.*	(a)	475
84 *Glycyrrhiza glabra*	Table 4	*Glycyrrhiza glabra L.*	(a)	475
85 *Gongronema latifolium*	Table 1	*Marsdenia latifolia* (Benth.) K.Schum.	(a)	4
86 *Gynotroches axillaris*	Table 1	*Gynotroches axillaris* Blume	(a)	1
87 *Hibiscus sabdariffa*	Table 1	*Hibiscus sabdariffa L.*	(a)	224
88 *Hoya kerrii*	Table 4	*Hoya kerrii Craib*	(a)	1
89 *Humulus japonicus*	Table 4	*Humulus scandens* (Lour.) Merr.	(a)	13
90 *Humulus lupulus*	Table 4	*Humulus lupulus L.*	(a)	159
91 *Hunteria zeylanica*	Table 4	*Hunteria zeylanica* (Retz.) Gardner ex Thwaite*s*	(a)	10
92 *Rhodocodon campanulatus*	Table 4	*Hyacinthaceae Sensu* APG II		3 (PubMed)
93 *Hypericum henryi*	Table 4	*Hypericum henryi* H.Lév. & Vaniot	(b)	4 (PubMed)
94 *Hypericum perforatum*	Table 1	*Hypericum perforatum L.*	(a)	220
95 *Hypericum perforatum*	Table 4	*Hypericum perforatum L.*	(a)	220
96 *Hyptis brevipes*	Table 4	*Hyptis brevipes* Poit.	(a)	5
97 *Impatiens balsamina*	Table 4	*Impatiens balsamina L.*	(a)	81
98 *Indigofera stachyodes*	Table 4	*Indigofera stachyodes* Lindl.	(b)	1
99 *Iris tectorum*	Table 4	*Iris tectorum* Maxim.	(a)	24
100 *Isodon pharicus*	Table 4	*Isodon pharicus* (Prain) Murata	(b)	6 (PubMed)
101 *Isodon scoparius*	Table 4	*Isodon scoparius* (C.Y.Wu & H.W.Li) H.Hara	(b)	7 (PubMed)
102 *Jatropha dioica*	Table 4	*Jatropha dioica Sessé*	(a)	14
103 *Jatropha gossypifolia*	Table 1	Jatropha gossypiifolia L.	(a)	16
104 *Juglans regia*	Table 1	*Juglans regia L.*	(a)	216
105 *Juglans regia*	Table 4	*Juglans regia L.*	(a)	216
106 *Justicia gendarussa*	Table 4	*Justicia gendarussa* Burm.f.	(a)	31
107 *Kopsia officinalis*	Table 4	*Kopsia arborea* Blume	(a)	0
108 *Laetia corymbulosa*	Table 4	*Laetia corymbulosa* Spruce ex Benth.	(b)	4 (PubMed)
109 *Lepidozia reptans*	Table 4	*Lepidozia reptans* (L.) Dumort.	(b)	2 (PubMed)
110 *Leplaea mayombensis*	Table 4	*Leplaea mayombensis* (Pellegr.) Staner	(a)	1
111 *Ligularia fischeri*	Table 4	*Ligularia fischeri* (Ledeb.) Turcz.	(a)	2

112	*Liquidambar formosana*	Table 4	*Liquidambar formosana* Hance	(a)	51
113	*Lithocarpus litseifolius*	Table 4	*Lithocarpus litseifolius* (Hance) Chun	(b)	2 (PubMed)
114	*Litsea cubeba*	Table 4	*Litsea cubeba* (Lour.) Pers.	(a)	56
115	*Macaranga tanarius*	Table 4	*Macaranga tanarius* (L.) Müll.Arg.	(a)	6
116	*Malus domestica*	Section 3.3	*Malus domestica* (Suckow) Borkh.	(a)	46
117	*Mammea harmandii*	Table 4	*Mammea harmandii* (Pierre) Kosterm.	(a)	1
118	*Millettia oblata*	Table 4	*Millettia oblata* Dunn	(a)	1
119	*Momordica balsamina*	Table 4	*Momordica balsamina L.*	(a)	69
120	*Momordica charantia*	Table 4	*Momordica charantia L.*	(a)	327
121	*Nauclea orientalis*	Table 4	*Nauclea orientalis* (L.) L.	(a)	7
122	*Ongokea gore*	Table 4	*Ongokea gore* (Hua) Pierre	(a)	14
123	*Onopordum acanthium*	Table 1	*Onopordum acanthium L.*	(a)	35
124	*Paganum harmal*	Table 1	*Peganum harmala L.*	(a)	94
125	*Panax ginseng*	Table 4	*Panax ginseng* C.A.Mey.	(a)	257
126	*Paramignya trimera*	Table 4	*Paramignya trimera* (Oliv.) Burkill	(b)	7 (PubMed)
127	*Paulownia tomentosa*	Table 4	*Paulownia tomentosa* Steud.	(a)	10
128	*Peganum harmala*	Table 4	*Peganum harmala L.*	(a)	94
129	*Pentalinon andrieuxii*	Table 4	*Pentalinon andrieuxii* (Müll.Arg.) B.F.Hansen & Wunderlin	(b)	12 (PubMed)
130	*Pentarhizidium orientale*	Table 4	*Onoclea orientalis* (Hook.) Hook.	(b)	3 (PubMed)
131	*Peperomia obtusifolia*	Table 4	*Peperomia obtusifolia (L.)* A.Dietr.	(a)	3
132	*Perovskia abrotanoides*	Table 4	*Perovskia abrotanoides* Kar.	(b)	19 (PubMed)
133	*Peucedanum japonicum*	Table 4	*Peucedanum japonicum* Thunb.	(a)	3
134	*Phaleria macrocarpa*	Table 1	*Phaleria macrocarpa* (Scheff.) Boerl.	(a)	1
135	*Phoradendron vernicosum*	Table 4	*Phoradendron wattii* Krug & Urb.	(a)	1
136	*Phyllanthus acidus*	Table 4	*Phyllanthus acidus (L.)* Skeels	(a)	66
137	*Phyllanthus flexuosus*	Table 4	*Phyllanthus flexuosus* (Siebold & Zucc.) Müll.Arg.	(b)	4 (PubMed)
138	*Physalis peruviana*	Table 4	*Physalis peruviana* L.	(a)	64
139	*Pistacia lentiscus*	Table 1	*Pistacia lentiscus L.*	(a)	99
140	*Plectranthus africanus*	Table 4	*Plectranthus africanus* (Baker) A.J.Paton	(a)	3
141	*Podocarpus nagi*	Table 4	*Podocarpus nagi* (Thunb.) Pilg.	(b)	19 (PubMed)

142	*Polygala flavescens*	Table 4	*Polygala flavescens* DC.	(b)	3 (PubMed)
143	*Polygonatum verticillatum*	Table 1	*Polygonatum verticillatum (L.)* All.	(a)	13
144	*Pongamia pinnata*	Table 4	*Pongamia pinnata (L.)* Pierre	(b)	356 (PubMed)
145	*Poupartia borbonica*	Table 4	*Poupartia borbonica* J.F.Gmel.	(a)	6
146	*Prangos haussknechtii*	Table 4	*Prangos asperula subsp. haussknechtii* (Boiss.) Herrnst. & Heyn	(a)	3 (PubMed)
147	*Pulicaria undulata*	Table 4	*Pulicaria undulata* (L.) C.A.Mey.	(a)	11
148	*Radula sumatrana*	Table 4	*Radula sumatrana* Stephani	(b)	1 (PubMed)
149	*Raphanus sativus*	Table 4	*Raphanus raphanistrum subsp. sativus* (L.) Domin	(a)	181
150	*Rhodocodon campanulatus*	Table 4	*Hyacinthaceae Sensu APG II*	(b)	3 (PubMed)
151	*Rhodomyrtus tomentosa*	Table 4	*Rhodomyrtus tomentosa* (Aiton) Hassk.	(a)	25
152	*Rumex nervosus*	Table 1	*Rumex nervosus* Vahl	(a)	3
153	*Salvia chamaedryoides*	Table 4	*Salvia chamaedryoides* Cav.	(b)	1 (PubMed)
154	*Salvia circinata*	Table 4	*Salvia circinnata* Cav.	(b)	1 (PubMed)
155	*Salvia miltiorrhiza*	Table 4	*Salvia miltiorrhiza* Bunge	(a)	89
156	*Salvia miltiorrhiza*	Section 1.4.1	*Salvia miltiorrhiza* Bunge	(a)	89
157	*Salvia plebeia*	Table 4	*Salvia plebeia* R.Br.	(a)	18
158	*Salvia polystachya*	Table 4	*Salvia polystachia* Cav.	(a)	1
159	*Sambucus williamsii*	Table 4	*Sambucus williamsii* Hance	(a)	15
160	*Satureja khuzistanica*	Section 3.1	*Satureja khuzistanica* Jamzad	(b)	35 (PubMed)
161	*Saxifraga spinulosa*	Table 4	*Saxifraga spinulosa* Adams	(b)	1 (PubMed)
162	*Schisandra bicolor*	Table 4	*Schisandra bicolor* W.C.Cheng	(b)	4 (PubMed)
163	*Schisandra chinensis*	Section 3.1	*Schisandra chinensis* (Turcz.) Baill.	(a)	118
164	*Scrophularia stiata*	Section 3.1	*Scrophularia striata* Boiss.	(a)	4
165	[illegible]*barbata*	Table 4	*Scutellaria barbata* D.Don	(a)	47
166	*Selaginella pulvinata*	Table 4	*Selaginella pulvinata* (Hook. & Grev.) Maxim.	(a)	27
167	*Siegesbeckia pubescens*	Table 4	*Sigesbeckia orientalis subsp. pubescens* (Makino) H.Koyama	(a)	21
168	*Sinningia reitzii*	Table 4	*Sinningia reitzii* (Hoehne) L.E. Skog	(b)	2 (PubMed)
169	*Siraitia grosvenorii*	Table 4	*Siraitia grosvenorii* (Swingle) C.Jeffrey ex A.M.Lu & Zhi Y.Zhang	(a)	37
170	*Sorbus acuparia*	Table 1	*Sorbus aucuparia L.*	(a)	39

171	*Streblus asper*	Table 4	*Streblus asper* Lour.	(a)	45
172	*Strychnos icaja*	Table 4	*Strychnos icaja* Baill.	(a)	27
173	*Tabernaemontana bufalina*	Table 4	*Tabernaemontana bufalina* Lour.	(a)	4
174	*Tagetes erecta*	Table 1	*Tagetes erecta L.*	(a)	94
175	*Taxus baccata*	Section 1.4.2	*Taxus baccata L.*	(a)	76
176	*Taxus brevifolia*	Section 1.4.2	*Taxus brevifolia* Nutt.	(a)	15
177	*Taxus wallichiana*	Table 4	*Taxus wallichiana* Zucc.	(a)	28
178	*Terminalia bentzoë*	Table 1	*Terminalia benzoe* Pers.	(b)	6 (PubMed)
179	*Terminalia macroptera*	Table 1	*Terminalia macroptera* Guill. & Perr.	(a)	41
180	*Teucrium yemense*	Table 4	*Teucrium yemense* Deflers	(b)	4 (PubMed)
181	*Thalictrum cultratum*	Table 4	*Thalictrum cultratum* Wall.	(a)	1
182	*Tinospora sagittata*	Table 4	*Tinospora sagittata* (Oliv.) Gagnep.	(a)	43
183	*Tinospora sinensis*	Table 4	*Tinospora sinensis* (Lour.) Merr.	(a)	34
184	*Trichospira verticillata*	Table 4	*Trichospira verticillata* (L.) S.F.Blake	(b)	1 (PubMed)
185	*Typhonium giganteum*	Table 4	*Typhonium giganteum* Engl.	(b)	17 (PubMed)
186	*Uncaria rhynchophylla*	Table 4	*Uncaria rhynchophylla* (Miq.) Miq. ex Havil.	(a)	57
187	*Urtica dioica*	Table 1	*Urtica dioica L.*	(a)	221
188	*Uvaria alba*	Table 4	*Uvaria alba* Merr. *(*unresolved)	(b)	1 (PubMed)
189	*Vaccinium ashei*	Table 4	*Vaccinium ashei* Reade	(b)	1198 (PubMed)
190	*Valeriana jatamansi Jones*	Section 2.3	*Valeriana jatamansi* Jones ex Roxb.	(a)	111
191	*Vallaris glabra*	Table 4	*Vallaris glabra* (L.) Kuntze	(b)	8 (PubMed)
192	*Verbesina lanata*	Table 4	*Verbesina lanata* B.L.Rob. & Greenm.	(b)	1 (PubMed)
193	*Vernonia amygdalina*	Table 1	*Vernonia amygdalina* Delile	(a)	96
194	*Vernonia cinerea*	Table 4	*Vernonia cinerea* (L.) Less.	(a)	76
195	*Vitex trifolia*	Table 4	*Vitex trifolia* L.	(a)	56
196	*Xylocarpus granatum*	Table 4	*Xylocarpus granatum* J.Koenig	(a)	12
197	*Xylocarpus rumphii*	Table 4	*Xylocarpus rumphii* (Kostel.) Mabb.	(b)	4
198	*Zephyranthes carinata*	Table 4	*Zephyranthes carinata* Herb.	(a)	1
199	*Zizyphus jujuba*	Table 4	*Ziziphus jujuba* Mill.	(a)	109
